# Objective technical skills assessment in laparoscopic cholecystectomy: a systematic review of manual, kinematic, and AI-based tools

**DOI:** 10.1007/s00464-026-13309-z

**Published:** 2026-08-31

**Authors:** Elizabeth M. A. Mainwaring, Nadia Guidozzi, Jonnie P. James, Jai Pantling, Nainika Menon, Riadh Salem, Sheraz R. Markar

**Affiliations:** 1 Surgical Intervention Trials Unit, Nuffield Department of Surgical Sciences, University of Oxford, Oxford, UK; 2 Faculty of Dentistry, Oral & Craniofacial Sciences, King’s College, London, UK; 3 School of Clinical Medicine, University of Cambridge, Cambridge, UK

**Keywords:** Laparoscopic cholecystectomy, Surgical skill assessment, Technical skills, Artificial intelligence, Validity evidence, Surgical education

## Abstract

**Background:**

Technical errors during surgery are a major contributor to preventable adverse outcomes, driving demand for objective assessment tools. Laparoscopic cholecystectomy (LC) is a commonly performed procedure with a clearly defined set of procedure-related complications, making it an ideal candidate for targeted assessment.

**Objective:**

To evaluate manual, kinematic, and AI-based technical skills assessment tools for LC and compare validity evidence and methodological quality.

**Methods:**

A PRISMA 2020-compliant systematic review (PROSPERO CRD420251125937) searched MEDLINE, Embase, Web of Science, and Cochrane Library from inception to 12 August 2025. Studies evaluating objective LC skill assessment tools were included. Two reviewers independently performed screening. Data extraction and study appraisal were performed with independent verification by a second reviewer. Validity was assessed using Messick’s framework, methodological quality using MERSQI, and risk of bias using COSMIN for manual and kinematic studies and QUADAS-2 for AI studies. Results were synthesised narratively.

**Results:**

Sixty studies were included (41 manual, 6 kinematic, 13 AI). Most were single centre and retrospective. Manual tools demonstrated the largest body of validity evidence across independent cohorts, though reliability was variable and outcome associations were lacking. Kinematic systems quantified motion but had limited validation. AI systems showed strong internal performance and early real-time use, particularly for critical view of safety detection, but lacked external validation and clinical impact evidence.

**Limitations:**

Heterogeneity precluded meta-analysis and limited assessment of reporting bias and certainty.

**Conclusions:**

No tool demonstrated sufficient validity to represent a gold standard. Manual tools are most mature but lack scalability, while AI systems show promise but require robust validation and evidence of clinical impact.

**Graphical abstract:**

**Figure:**
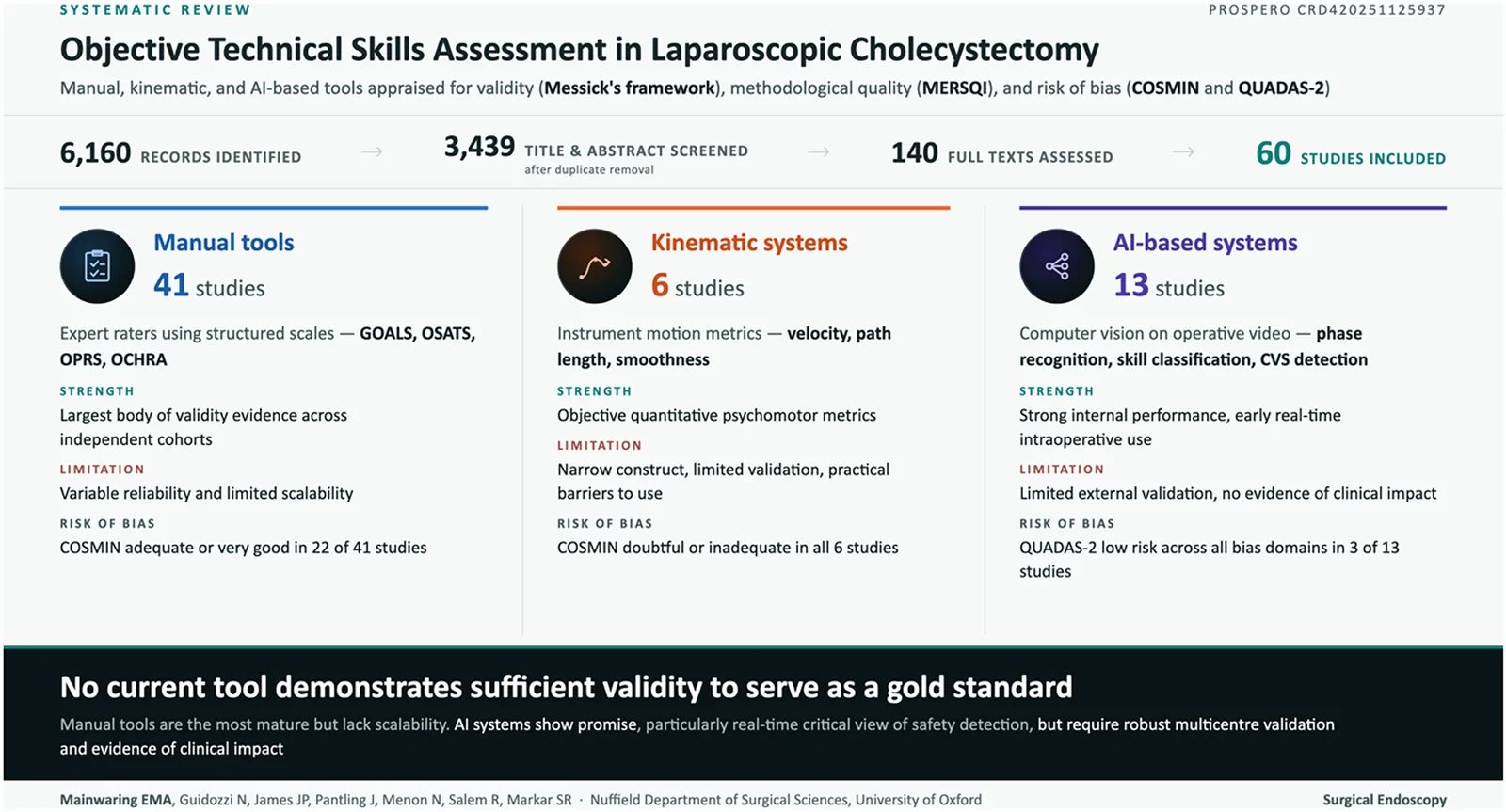

**Supplementary Information:**

The online version contains supplementary material available at https://doi.org/10.1007/s00464-026-13309-z.

Technical errors during surgery are a major contributor to preventable patient harm. Surgical complications account for approximately 40% of hospital adverse events; separately, approximately 44% of all adverse events are considered preventable. Adverse events are associated with substantial healthcare costs through prolonged hospitalisation, intensive care utilisation, and litigation [1–3]. Intraoperative technical errors are particularly associated with increased post-operative morbidity, and thus must be reliably analysed to provide surgeons with feedback to support surgical training and learning [4, 5].

The shift from an apprenticeship model towards competency-based surgical training has created a need for objective, reliable, quantitative measures of operative performance to support safe trainee progression and high-quality care [6]. Structured assessment frameworks have been associated with improved patient outcomes [7] and are increasingly important not only for training, but also for research, where standardised measures enable comparison of technical performance across studies and centres [8].

Objective technical skills assessment tools can be broadly categorised into three modalities: manual observational tools, kinematic systems, and artificial intelligence (AI)-based approaches. Manual tools rely on expert raters using structured scales, including global rating scales, error-based assessments, and procedural-specific checklists. Kinematic systems quantify instrument motion characteristics, such as velocity and path length, as proxies for technical skill. More recently, AI-based approaches represent an emerging frontier, leveraging computer vision and machine learning to analyse operative video and automate performance evaluation [9].

Laparoscopic cholecystectomy (LC) provides an ideal model for evaluating such tools. It is one of the most commonly performed general surgical procedures in the UK, with over 73,000 procedures undertaken in England in 2023–2024 [10]. Given that LC has standardised steps, a high case volume, and well-defined safety criteria, it serves as a robust index procedure for evaluating competency-based assessment tools and benchmarking trainee surgical performance.

Evaluation of surgical assessment tools requires a robust framework for validity. Messick’s unified theory of validity [11] incorporates multiple sources of evidence to provide a comprehensive evaluation of assessment quality, including content, response process, internal structure, generalisability, relations to other variables, and consequences, each defined in Supplementary Table S1. This framework provides a comprehensive assessment of measurement quality and is widely adopted in medical education research.

While competency-based assessment has been widely explored, existing systematic reviews have either examined surgical technical-skill assessment across procedures or focussed on a single assessment modality. Grüter et al. synthesised global, error-based, procedural-specific, and AI-based assessment tools across laparoscopic surgery [8], while Lam et al. reviewed machine-learning-based approaches to surgical-skill assessment [12]. However, no previous review has synthesised manual, kinematic, and AI-based tools specifically for laparoscopic cholecystectomy or compared their validity evidence using a common Messick framework.

This systematic review aims to evaluate manual, kinematic, and AI-based technical skills assessment tools for laparoscopic cholecystectomy, comparing the strength of validity evidence and methodological quality. Secondary aims are to identify candidate benchmark tools for emerging technologies and to assess their readiness for integration into surgical training.

## Methods

### Protocol and reporting

This systematic review is reported in accordance with the Preferred Reporting Items for Systematic Reviews and Meta-Analyses (PRISMA) 2020 statement [13]. Minor deviations from the protocol were made. Specifically, planned outcome measures including OCEBM levels of evidence and GRADE assessment were not applied, as preliminary data extraction demonstrated substantial heterogeneity in study design and outcome reporting, limiting the feasibility and interpretability of these frameworks.

### Eligibility criteria

Eligibility was defined using the Population, Intervention, Comparison, Outcome (PICO) framework:

- Population: Surgical trainees or consultants assessed on their performance in laparoscopic cholecystectomy.
- Intervention: Objective technical skills assessment tool or method.
- Comparison: Alternative assessment tools, human raters, or benchmarked evaluation methods.
- Outcomes: Measures of validity, reliability, accuracy, and clinical impact.

Inclusion criteria were comprised of original research, validation studies, and methodological papers evaluating observational assessment of technical skills in laparoscopic cholecystectomy. Exclusion criteria included (i) studies limited to open or robotic or other laparoscopic procedures without specific application to laparoscopic cholecystectomy; (ii) studies lacking a defined assessment or scoring system; (iii) those focussing exclusively on non-technical skills; (iv) opinion pieces, editorials, conference abstracts without full text, or papers unavailable in English; and (v) studies using virtual reality, simulation, or animal models without direct operative application. Review articles were not included in the synthesis but were examined for potentially eligible primary studies.

### Search strategy

A comprehensive search was developed in consultation with a Bodleian Health Care Sciences librarian. Searches were conducted in MEDLINE, Embase, Web of Science, and the Cochrane library from inception to the 12th of August 2025. Search terms combined keywords and MeSH terms for “laparoscopic cholecystectomy”, “competence”, “skill”, and “assessment” using Boolean operators. Reference lists of included studies were manually screened for additional eligible articles. Full search strategies for each database are provided in the Supplementary Methods.

### Study selection and data extraction

**Fig. 1:**
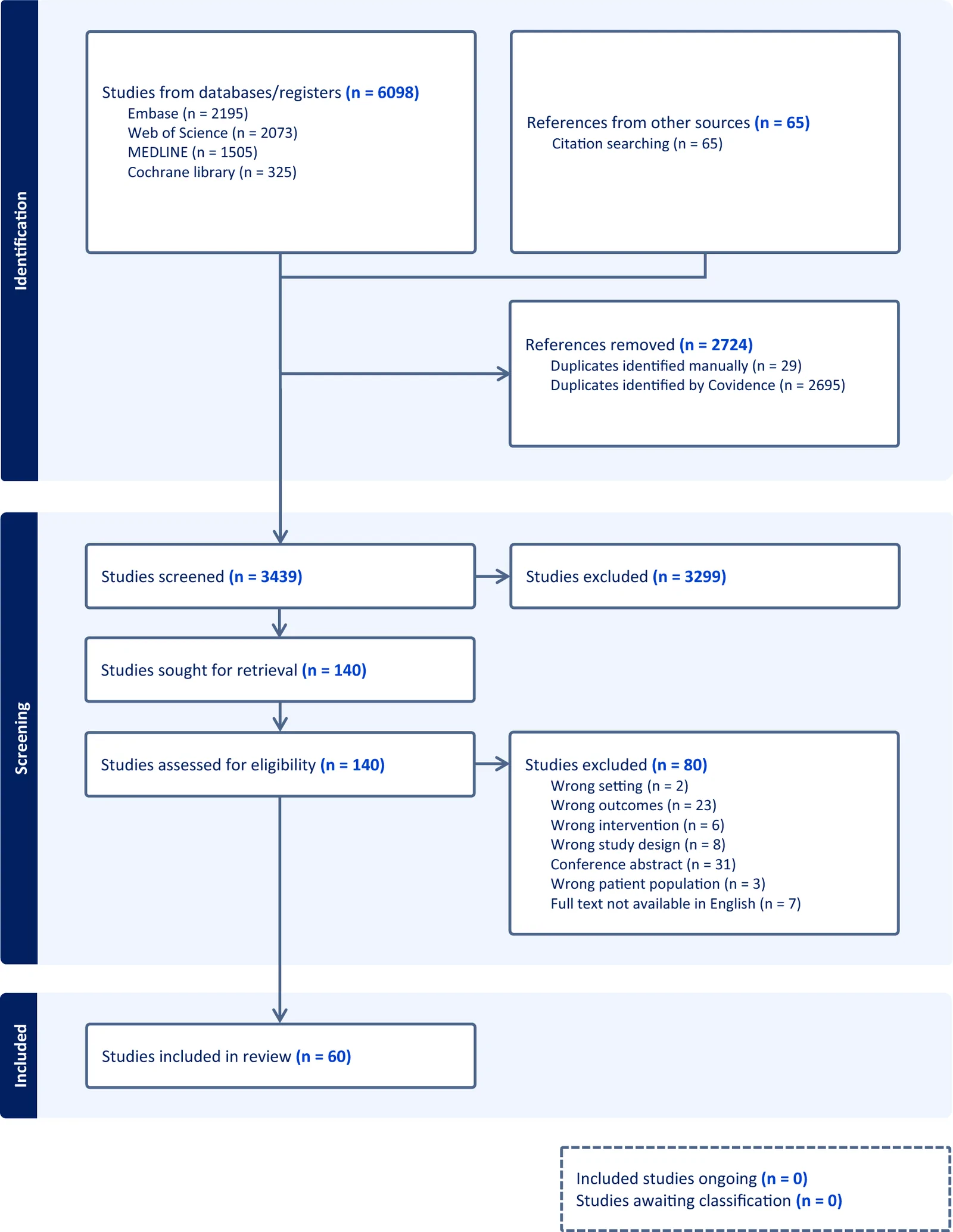
PRISMA flow diagram of study selection for inclusion in the systematic review. A total of 6098 records were identified through database and registry searching (Embase, Web of Science, MEDLINE, Cochrane library, and citation searching), with an additional 65 records identified through other sources. After removal of 2724 duplicates, 3439 records underwent title and abstract screening, of which 3299 were excluded. A total of 140 full-text articles were assessed for eligibility, with 80 excluded for the following reasons: wrong setting (*n* = 2), wrong outcomes (*n* = 23), wrong intervention (*n* = 6), wrong study design (*n* = 8), conference abstract only (*n* = 31), wrong patient population (*n* = 3), and full text not available in English (*n* = 7). Sixty studies met the inclusion criteria and were included in the final synthesis

Search results were imported into Covidence for automatic de-duplication and screening. Studies underwent initial title and abstract screening, followed by full-text screening, by at least two independent reviewers (EMAM, JJ, and JP), with all conflicts resolved through discussion and consensus. Study selection is summarised in the PRISMA flow diagram: see Fig. 1.

Data extraction was performed by one reviewer and independently verified by a second, with discrepancies resolved through discussion and consensus. Extracted variables included publication year, country, number of participating institutions, assessment tool name and classification (manual, kinematic, or AI-based), number and experience of operators, number and experience of assessors, assessor blinding, and assessor training. Data were extracted into a pre-piloted spreadsheet to ensure consistency across studies.

### Data synthesis, assessment of validity, methodological quality, and bias

Due to heterogeneity in study designs, populations, outcome measures, and assessment frameworks, no quantitative effect measures were calculated, and meta-analysis was not performed. Results were synthesised using a structured narrative approach, with studies grouped by assessment modality (manual, kinematic, and artificial intelligence-based tools) and summarised descriptively across Messick’s validity domains. No formal assessment of statistical heterogeneity, reporting bias, or certainty of evidence was undertaken.

Two independent blinded reviewers extracted all information relating to scoring systems, with discrepancies resolved by consensus decision. Each study’s validity evidence was appraised using Messick’s framework [11] for construct validity, comprising six domains: content, response process, internal structure, relations to other variables, consequences, and generalisability. Each domain was graded as *reported-limited*, *reported-partial*, *reported-strong*, or *not reported*, based on study methodology and reporting quality: Supplementary Table S1. Methodological quality was assessed using the Medical Education Research Study Quality Instrument (MERSQI) [14].

Studies were considered suitable for quantitative pooling only where they evaluated sufficiently comparable populations, assessment constructs, tools, outcome measures and units of analysis. Meta-analysis was not undertaken where studies differed materially in these domains such that a pooled estimate would not have a clinically or methodologically coherent interpretation.

Two independent assessors (NM and NG) undertook quality assessments for all included studies, and any disagreements were resolved by a third assessor (LM). The Quality Assessment of Diagnostic Accuracy Studies (QUADAS-2) tool was used to qualitatively appraise 13 diagnostic accuracy (AI) studies [15]. As per QUADAS-2 rating criteria, each study was scored ‘high risk’, ‘low risk’, or ‘unclear’ across the ‘Risk of bias’ and ‘Applicability concerns’ domains. The COnsensus-based Standards for the selection of health Measurement INstruments (COSMIN) tool was used to qualitatively appraise 41 manual-assessment tools-based studies and 6 kinematic systems-based studies [16]. Each domain was scored on a 4-point scale: ‘very good’, ‘adequate’, ‘doubtful’, and ‘inadequate’.

### Use of artificial intelligence

ChatGPT (OpenAI, San Francisco, CA, USA) was used to assist with manuscript editing, structuring, and visual conceptualisation. No AI tools were used for data extraction, screening, or statistical analysis. All interpretations and conclusions were made by the authors.

## Results

### Study characteristics

A comprehensive literature search identified 6098 records through database searching and 65 additional records through reference screening. After removal of duplicates (*n* = 2724), 3439 records underwent title and abstract screening, of which 3299 were excluded based on predefined eligibility criteria. A total of 140 full-text articles were assessed for eligibility, and 80 were excluded, most commonly due to conference abstract-only publication (*n* = 31), inappropriate outcome measures (*n* = 23), or unsuitable study design (*n* = 8). Sixty studies met the inclusion criteria and were included in the final synthesis (Fig. 1) [17–76]. Studies excluded at full-text screening with reasons is supplied in Supplementary Table S2.

Of the included studies, 41 evaluated manual-assessment tools [18–20, 22–25, 28, 31–34, 37, 39, 40, 42–46, 49, 53–71, 76], 6 examined kinematic systems [21, 26, 27, 29, 30, 36], and 13 investigated AI-based methods [17, 35, 38, 41, 47, 48, 50–52, 72–75]. Across paradigms, reporting of patient characteristics was inconsistent. Where specified, studies predominantly included elective, uncomplicated laparoscopic cholecystectomy cases. However, operative setting and case mix were frequently unreported. A minority of studies included mixed elective and emergency cohorts or acute cholecystitis cases.

Approximately two-thirds of studies were conducted in single centres, and the majority (43/60, 71.7%) were retrospective in design. Only 3 AI-based systems were deployed intraoperatively in real time [41, 48, 51], and none of the assessment tools evaluated downstream clinical or patient safety outcomes. Sample sizes varied substantially, ranging from 2 to 431 procedures.

Detailed per-study results are provided in Supplementary Table S3**.** Substantial methodological heterogeneity was present across the three assessment modalities, as displayed in Supplementary Table S4, with differences in study populations, assessment methods, units of analysis, and reported outcomes. The studies therefore did not provide sufficiently comparable estimates for a clinically meaningful meta-analysis.

### Manual-assessment tools

Manual-assessment tools were grouped into global rating scales, procedural-based assessment tools, technical error-based assessment scales, and hybrid tools, combining both a global and procedural-based assessment. The 41 manual-assessments studies contributed to 46 study-tool- evaluations because some studies evaluated more than one assessment instrument or category. Global rating scales represented the most frequently evaluated subgroup (23 studies), followed by procedural-based tools (14 studies), and technical error-based scales (8 studies), while only a single hybrid instrument was identified. Global Operative Assessment of Laparoscopic Skills (GOALS) and Objective Structured Assessment of Technical Skills (OSATS) were the most commonly used global rating scales, with Operative Performance Rating System (OPRS) and Observational Clinical Human Reliability Analysis (OCHRA) representing the most frequently evaluated procedural-based and error-based tools, respectively. Characteristics of included tools and the studies evaluating them, including patient populations, operative components assessed, and timing of assessment, are summarised in Table 1. Evidence supporting validity across Messick’s framework is summarised at the tool level in Table 2.

**Table 1:** Characteristics of manual technical skills assessment tools and their evaluation contexts in laparoscopic cholecystectomy

Tool	No. of studies	Total procedures	Population	Operative component assessed	Timing of assessment
Global rating scales					
GOALS	9	253	Predominantly elective uncomplicated LC; population not reported in most studies	Predominately whole operation; Calot’s triangle dissection also assessed	Predominantly video-based retrospective assessment; some live intraoperative and post-operative direct recall
OSATS	7	398	Predominantly elective uncomplicated LC; population not reported in most studies	Whole operation	Predominantly video-based retrospective assessment; some live intraoperative and post-operative direct recall
mOSATS	3	177	Predominantly elective uncomplicated LC	Whole operation	Predominantly video-based retrospective assessment; some post-operative direct recall
OpRate	1	142	Not reported	Whole operation	Post-operative direct recall
mLCAT	1	42	Mixed elective/emergency LC	Whole operation	Live and retrospective
OSA‐CNS	1	24	Not reported	Whole operation	Post-operative direct recall
SALAS	1	20	Elective uncomplicated LC	Whole operation	Video-based retrospective assessment
Procedural-based assessment scales					
Operative Performance Rating System (OPRS)	5	720	Mixed elective/emergency LC; population not reported in the majority	Whole operation	Combination of video-based retrospective assessment and post-operative direct recall and live intraoperative assessment
Procedure-specific checklist by Sarker et al.	3	137	Predominantly elective uncomplicated LC	Whole operation	Video-based retrospective assessment
LC-CVS OPSA	2	45	Varying case difficulty; operative setting (elective vs. emergency) not reported	Calot’s triangle dissection	Video-based retrospective assessment
Strasberg CVS crtieria	1	100	Mixed elective/emergency LC	Calot's triangle dissection	Photograph-based retrospective assessment
ISCP PBA	1	84	Elective uncomplicated LC	Whole operation	Video-based retrospective assessment
PBA (Isreb et al.)	1	10	Not reported	Whole operation	Combination of video-based retrospective assessment and post-operative direct recall
PBA (van Zwieten et al.)	1	35	Elective uncomplicated LC	Whole operation	Post-operative direct recall
Hybrid scales					
OPR	1	2	Elective uncomplicated LC	Whole operation	Combination of video-based retrospective assessment and live intraoperative assessment
Technical error-based assessment scales					
OCHRA	2	208	Elective uncomplicated LC	Whole operation	Video-based retrospective assessment
LC technical error-based tool (Sarker et al.)	2	137	Elective uncomplicated LC	Whole operation	Video-based retrospective assessment
LC technical error-based tool (Eubanks et al.)	1	30	Not reported	Whole operation	Video-based retrospective assessment
LC technical error-based tool (Joice et al.)	1	20	Not reported	Whole operation	Video-based retrospective assessment
LC technical error-based tool (Seymour et al.,)	1	14	Not reported	Excisional phase of lap chole, after division of the cystic structures	Video-based retrospective assessment
LC technical error-based tool (Kramp et al.)	1	3	Not reported	Whole operation	Video-based retrospective assessment

Manual tools are presented by assessment category (global rating scales, procedural-based assessment scales, hybrid scales, and technical error-based assessment scales). For each instrument, the table summarises the number of evaluating studies, cumulative number of procedures, study population, operative component assessed, and timing of assessment

*LC* laparoscopic cholecystectomy, *CVS* critical view of safety, *OSATS* Objective Structured Assessment of Technical Skills, *mOSATS* modified Objective Structured Assessment of Technical Skills, *GOALS* Global Operative Assessment of Laparoscopic Skills, *mLCAT* modified Laparoscopic Cholecystectomy Assessment Tool, *OSA-CNS* Objective Structured Assessment of Critical Node Skills, *SALAS* Structured Assessment of Laparoscopic Assistant Skills, *OPRS* Operative Performance Rating System, *PBA* Procedure-Based Assessment, *OPSA* Operative Performance Scoring Algorithm, *OCHRA* Observational Clinical Human Reliability Analysis

**Table 2:**
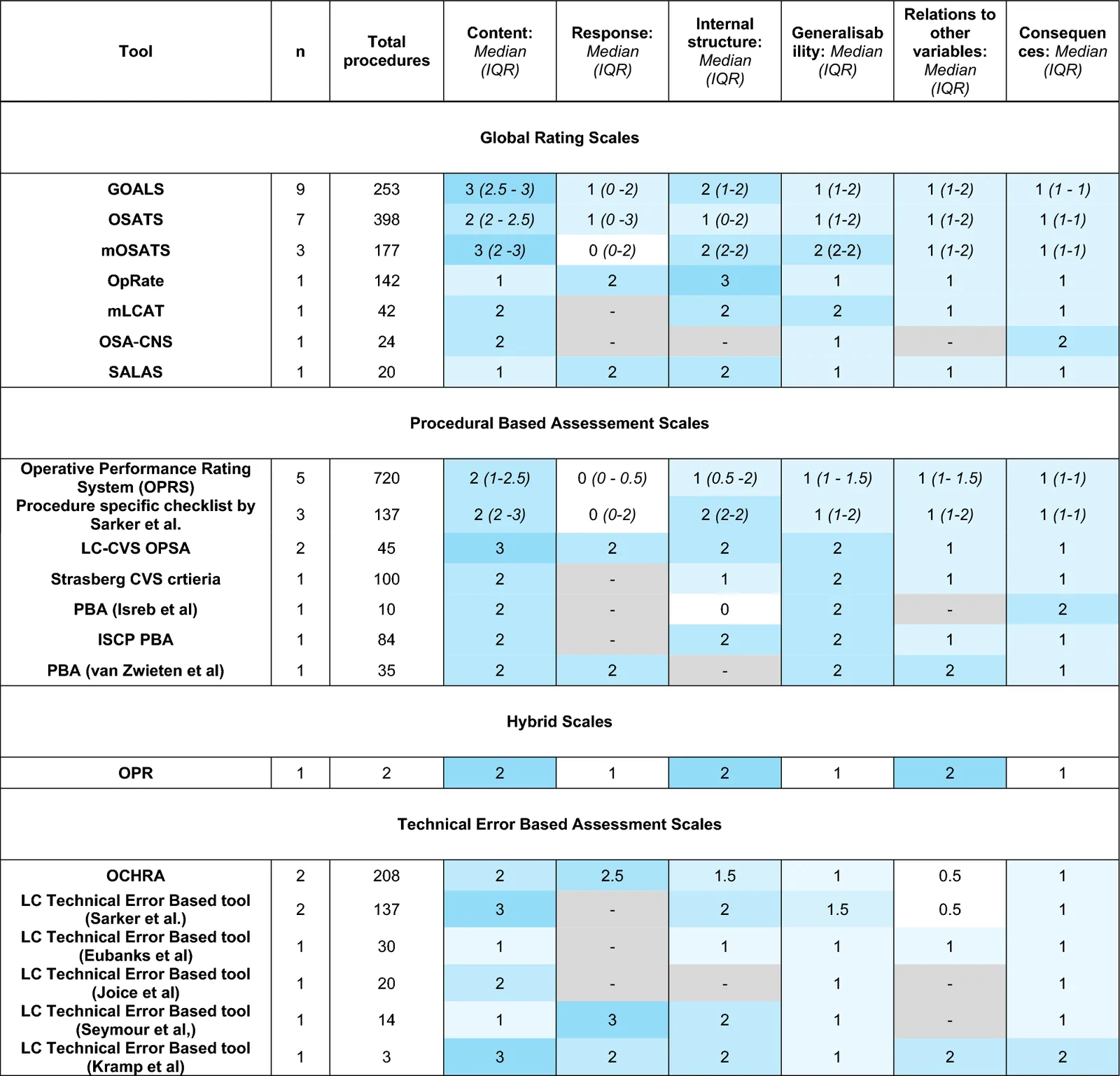
Tool-level validity evidence for manual technical skills assessment instruments used in laparoscopic cholecystectomy

Manual tools are grouped by assessment category. For each instrument, the table summarises the number of evaluating studies, cumulative number of procedures, and median domain-specific validity evidence ratings across Messick’s framework, reported as median (interquartile range) where more than two studies were available. Ratings were assigned using the evidence rubric shown in Supplementary Table S1. Domains include content, response process, internal structure, generalisability, relations to other variables, and consequences. A dash indicates that no data were available for that domain

*LC* laparoscopic cholecystectomy, *CVS* critical view of safety, *OSATS* Objective Structured Assessment of Technical Skills, *mOSATS* modified Objective Structured Assessment of Technical Skills, *GOALS* Global Operative Assessment of Laparoscopic Skills, *mLCAT* modified Laparoscopic Cholecystectomy Assessment Tool, *OSA-CNS* Objective Structured Assessment of Critical Node Skills, *SALAS* Structured Assessment of Laparoscopic Assistant Skills, *OPRS* Operative Performance Rating System, *PBA* Procedure-Based Assessment, *OPSA* Operative Performance Scoring Algorithm, *OCHRA* Observational Clinical Human Reliability Analysis

The majority (34/41 manual-assessment studies, 82.9%) of studies evaluated technical performance across the whole operation. Among the nine studies evaluating GOALS, three (33.3%) assessed selected operative phases rather than the entire procedure [44, 68, 69]. Two of the seven procedural-based instruments focussed specifically on Calot’s triangle dissection and attainment of the CVS [18, 19]. One of eight technical error-based study–tool evaluations assessed a defined operative phase [61]. Assessment timing varied across tools but was predominantly video-based and retrospective. Six of the 41 studies (14.6%) incorporated mixed approaches, including live intraoperative assessment and post-operative direct recall, with only 7 of 41 studies (17.1%) relying exclusively on immediate post-operative evaluation.

Across Messick’s framework, manual tools demonstrated moderate evidence for content validity, which reflects alignment with established surgical competency frameworks and consensus-defined operative standards. Response process evidence on balance was limited or unreported, with many studies not reporting rater training or calibration in their methodology. Internal structure evidence was heterogeneous, while some studies reported inter-rater reliability metrics, others relied on descriptive agreement alone. Generalisability was limited by small populations, most frequently capturing uncomplicated elective cases, and therefore not representative of the breadth of pathology and potential technical difficulty of LC. Relations to other variables were typically examined through correlations with trainee level or through comparison to another manual-assessment tool. No study evaluated associations between assessment scores and patient outcomes, and consequential validity evidence was largely limited to speculation of potential positive effect on trainee progression.

### Kinematic assessment tools

Six studies evaluated kinematic motion-modelling systems using electromagnetic tracking, optical sensors, or two-dimensional video-based motion analysis [21, 26, 27, 29, 30, 36]. Sample sizes ranged from 6 to 47 procedures. Study characteristics, including patient populations, operative components assessed, and timing of assessment, are summarised in Table 3. Evidence supporting validity across Messick’s framework is summarised at the tool level in Table 4.

**Table 3:** Characteristics of kinematic systems and their evaluation contexts in laparoscopic cholecystectomy

Tool	No. of studies	Total procedures	Population	Inputs	Outputs	Operative component assessed	Timing of assessment
MCMD-based intraoperative kinematic assessment	1	18	Not reported	3D electromagnetic instrument tracking; video-derived gesture labels; kinematic features (velocity, acceleration, jerk)	Velocity, jerk, transition probabilities	Calot’s triangle exposure and cystic duct/artery dissection	Intraoperative data capture with retrospective analysis
Multimodal motor performance (MMP) system	1	6	Biliary colic	Optical + electromagnetic instrument tracking (3D); kinematic metrics; force/torque sensors; video error scoring	Combined motion and force metrics	Instrument-specific analysis (modified Maryland dissector)	Intraoperative data capture with retrospective analysis
ROVIMAS video-based motion tracking device	2	57	Mixed case difficulty but predominantly biliary colic	Electromagnetic hand-motion tracking (3D); synchronised laparoscopic video; OSATS rating	Time, path length, movement count	Whole operation	Video-based retrospective assessment
Kinovea motion tracking system	1	29	Uncomplicated LC	2D laparoscopic video; manual instrument-tip tracking	Path length, movement speed, extreme movements	Whole operation	Video-based retrospective assessment
Kinovea + weighted threshold algorithm	1	12	Uncomplicated LC	2D laparoscopic video; instrument-tip tracking; derived motion metrics	Continuous performance score (0–1); expertise classification	Whole operation	Video-based retrospective assessment

For each system, the table summarises the number of evaluating studies, cumulative number of procedures, study population, input data streams, derived outputs, operative component assessed, and timing of assessment

*LC* laparoscopic cholecystectomy, *OSATS* Objective Structured Assessment of Technical Skills, *MCMD* Markov chain-based motion decomposition, *MMP* multimodal motor performance

**Table 4:**
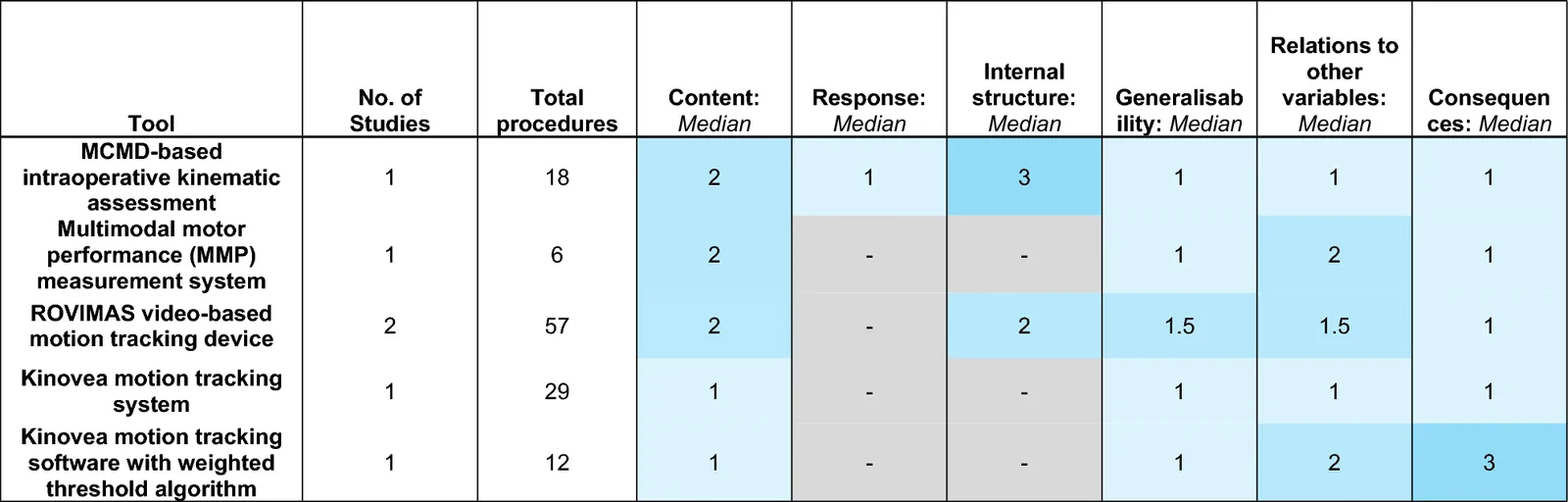
Tool-level validity evidence for kinematic systems used to assess technical performance in laparoscopic cholecystectomy

For each kinematic system, the table summarises the number of evaluating studies, cumulative number of procedures, and domain-specific validity evidence ratings across Messick’s framework. Ratings are presented as median values. Interquartile ranges were not calculated as no system was evaluated in more than two studies. Domains include content, response process, internal structure, generalisability, relations to other variables, and consequences. A dash indicates that no data were available for that domain

*LC* laparoscopic cholecystectomy, *OSATS* Objective Structured Assessment of Technical Skills, *MCMD* Markov chain-based motion decomposition, *MMP* multimodal motor performance

These systems quantified instrument movement characteristics, including velocity, acceleration, jerk, path length, and movement smoothness as a proxy for surgical technical skills. Only one system incorporated force/torque measurements [36]. Data acquisition was frequently performed intraoperatively in real time, although analysis was typically conducted retrospectively and did not inform live decision-making.

Content validity was moderate, reflecting plausible alignment between psychomotor efficiency and surgical performance. The majority did not provide evidence of response or internal validity, with formal reliability testing also being limited. Generalisability was constrained by small datasets and, in some cases, restriction to specific instruments or operative steps. Relations to other variables were limited to moderate, with some systems demonstrating discrimination between expertise levels using derived motion parameters. However, no study evaluated clinical outcomes. Consequential validity was limited with only one study establishing a threshold score for acceptable versus unacceptable performance.

### AI-based assessment tools

13 studies evaluated AI-based systems, which can be grouped into three construct-driven categories: (1) phase and workflow recognition systems [35, 52, 73], (2) direct skill classification models [17, 47, 74, 75], and (3) safety–critical construct detection systems focussed on the critical view of safety (CVS) [38, 41, 48, 50, 51, 72]. Model architectures included convolutional neural networks, transformer-based video models, classical machine learning classifiers, and hybrid deep learning frameworks incorporating temporal modelling, object detection, and semantic segmentation techniques. Study characteristics, including patient populations, training and test data sets, and timing of assessment, are summarised in Table 5. Evidence supporting validity across Messick’s framework is summarised at the tool level in Table 6.

**Table 5:** Characteristics of artificial intelligence-based systems and their evaluation contexts in laparoscopic cholecystectomy

Tool	No. of studies	Total procedures	Population	Model paradigm	Training set	Test set	Inputs	Outputs	Operative component assessed	Timing of assessment
Phase and workflow recognition systems										
Humm (Touch Surgery)	1	159	Elective LC	Deep learning phase recognition	Not reported	159 LC videos	Laparoscopic video frames	Phase labels + phase durations	Whole operation	Video-based retrospective assessment
Pradeep (SC-CNN + SSM)	1	10	Cholec80 + m2cai16	CNN +temporal modelling	64 Cholec80 videos	16 Cholec80 + 10 m2cai16 videos	Laparoscopic video frames	Phase labels + continuous skill score	Whole operation	Video-based retrospective assessment
Yanagida (EfficientNetB7)	1	86	Acute or chronic cholecystitis	CNN image classifier	150 videos	38 validation + 50 test	Laparoscopic video frames	10-phase classification with phase time metrics	Whole operation	Video-based retrospective assessment
Direct skill classification										
Chen (SVM Surgesture)	1	75	Uncomplicated acute and chronic cholecystitis	Classical ML model	5-fold cross-validation	1/5 split per fold (~ 15 videos)	Surgesture-derived features	Competent vs. incompetent classification	Calot’s triangle dissection	Video-based retrospective assessment
Lavanchy (CNN + regression)	1	~30% of 944 segments	Not documented	CNN + regression model	70% of segments	30% of segments	Laparoscopic video frames of clip application	Predicted skill rating (1–5) + binary skill classification	Clipping of cystic artery and duct	Video-based retrospective assessment
Yen (VideoMAE + RF)	1	14	Chole80	Transformer (VideoMAE) + Random Forest	66 LC videos	14 LC videos	Laparoscopic video frames	Action recognition + competency prediction	Calot’s triangle dissection	Video-based retrospective assessment
Yanik (A-VBANet)	1	15	Not reported	CNN meta-learning mode	Simulator + JIGSAWS tasks + unlabeled LC videos	OSATS labelled LC videos	Laparoscopic video frames	Task-level skill classification	Basic laparoscopic tasks	Video-based retrospective assessment
Safety–critical construct detection (CVS-focussed systems)										
Mascagni (DeepCVS)	1	201	Elective LC	CNN segmentation + classifier	60% images (5-fold CV)	20% images per fold	Laparoscopic video images	Multi-label prediction of CVS criteria + segmentation	Critical view of safety (CVS)	Video-based retrospective assessment
Kawamura (EfficientNet-B5)	1	20	Uncomplicated acute cholecystitis	CNN classifier	18,000 annotated images	20 LC videos	Laparoscopic video images	Binary prediction of CVS components	Critical view of safety (CVS)	Real-time automated assessment
Leifman (SpineNet + ResNet)	1	40	Biliary colic, acute and chronic cholecystitis	CNN segmentation + classifier	560 LC videos	140 LC videos + 40 prospective	Laparoscopic video frames	CVS achievement prediction	Critical view of safety (CVS)	Real-time automated assessment
Petracchi (YOLOv8)	1	40	Elective LC or uncomplicated acute cholecystitis	Object detection network	402 annotated images	40 LC cases	Laparoscopic video frames	Detection of cystic duct/artery and CVS inference	Critical view of safety (CVS)	Real-time automated assessment
Wu (SurgSmart)	1	431	Multicentre prospective dataset (38% severe inflammation)	Hybrid deep learning system	676 LC videos	431 LC videos	Laparoscopic video frames	Phase recognition, CVS scoring, disease severity	Critical view of safety (CVS)	Video-based retrospective assessment
Jin (tool detection system)	1	5	m2cai16	Faster R-CNN	10 LC videos	5 LC videos	Laparoscopic video frames	Tool detection with derived safety metrics	Critical view of safety (CVS)	Video-based retrospective assessment

AI tools are grouped by construct category, including phase and workflow recognition systems, direct skill classification models, and safety–critical construct detection systems. For each tool, the table summarises the number of evaluating studies, total procedures, study population, model paradigm, training and test sets, input data, outputs, operative component assessed, and timing of assessment

*LC* laparoscopic cholecystectomy, *CVS* critical view of safety, *CNN* convolutional neural network, *SVM* support vector machine, *RF* random forest, *SC-CNN* spatial–channel convolutional neural network, *SSM* state-space model, *VideoMAE* Video Masked Autoencoder, *A-VBANet* Attention-based Video-Based Assessment Network, *YOLO* You Only Look Once, *R-CNN* region-based convolutional neural network, *ResNet* residual neural network, *SpineNet* scale-permuted backbone network, *OSATS* Objective Structured Assessment of Technical Skills, *JIGSAWS* JHU-ISI Gesture and Skill Assessment Working Set; *Cholec80* 80-case laparoscopic cholecystectomy video dataset, *m2cai16* 2016 MICCAI workflow challenge dataset *CV* cross-validation

**Table 6:**
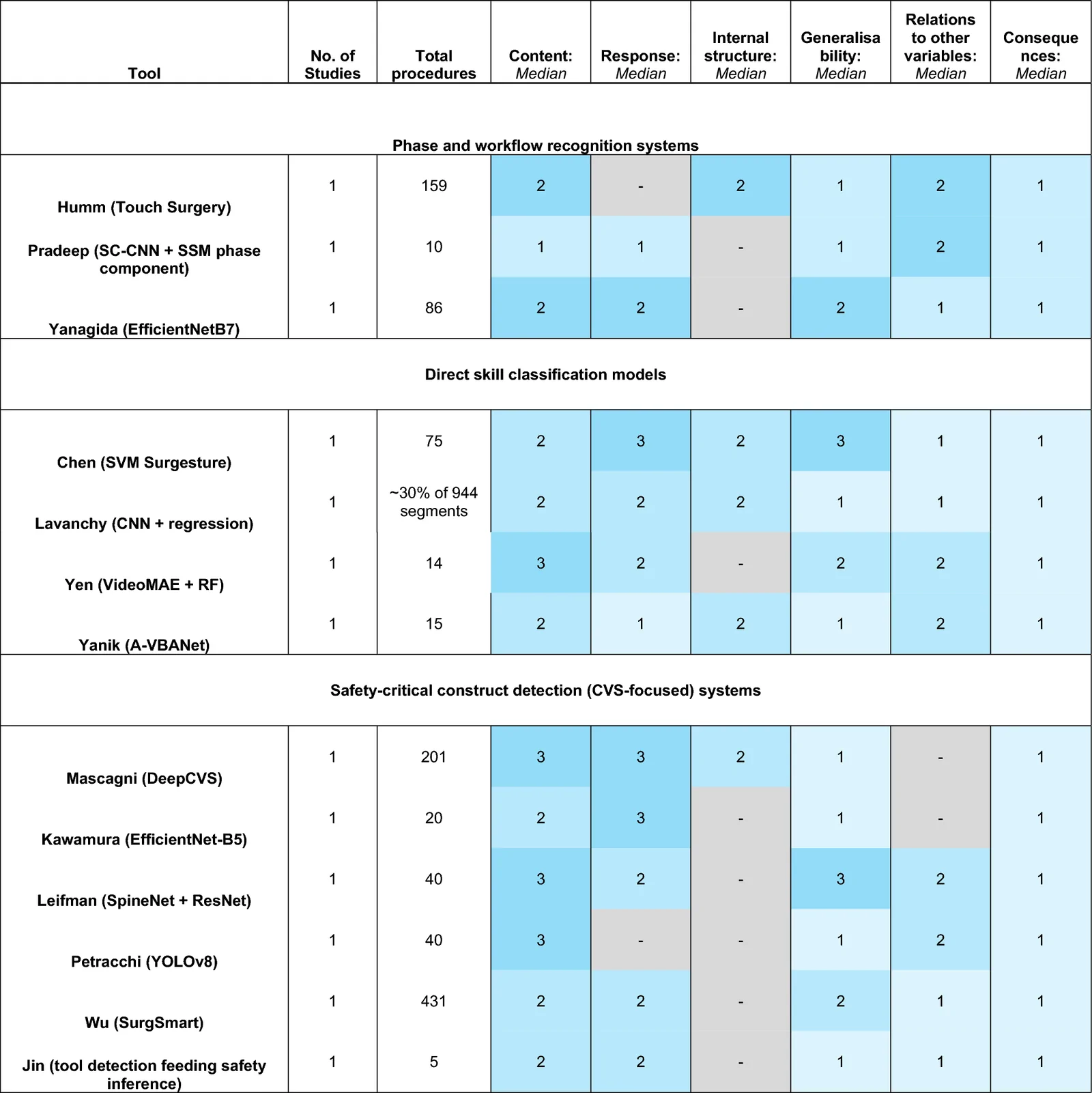
Tool-level validity evidence for artificial intelligence-based systems used to assess technical performance in laparoscopic cholecystectomy

AI systems are grouped by construct category, including phase and workflow recognition systems, direct skill classification models, and safety–critical construct detection systems. For each tool, the table summarises the number of evaluating studies, cumulative number of procedures, and domain-specific validity evidence ratings across Messick’s framework. Ratings are presented as median values. Interquartile ranges were not calculated as no system was evaluated in more than two studies. Domains include content, response process, internal structure, generalisability, relations to other variables, and consequences. A dash indicates that no data were available for that domain

*IQR* interquartile range, *CVS* critical view of safety, *CNN* convolutional neural network, *SVM* support vector machine, *RF* random forest, *SC-CNN* spatial–channel convolutional neural network, *SSM* state-space model, *VideoMAE* Video Masked Autoencoder, *A-VBANet* Attention-based Video-Based Assessment Network, *YOLO* You Only Look Once, *ResNet* residual neural network, *SpineNet* scale-permuted backbone network

AI systems were predominantly (12/13 AI studies, 92.3%) developed using laparoscopic video data, with only 1 study incorporating multimodal inputs [72]. Study sample sizes ranged from 5 to 431 procedures. Most studies were retrospective in design, with only 3 systems demonstrating real-time intraoperative capability [41, 48, 51], including 2 with prospective validation cohorts [48, 72]. Outputs varied according to the underlying construct, including multi-class phase labels, temporal workflow metrics, binary or continuous skill scores, and detection of safety–critical operative endpoints such as CVS attainment.

Across Messick’s framework, content validity was generally moderate to strong, especially in the CVS-focussed systems, reflecting clear alignment with internationally established critical view of safety definitions. Response process validity was variably reported, with CVS-focussed systems again demonstrating more robust evidence, reflecting structured annotation protocols and ground-truth labelling processes, whereas phase recognition and skill classification models often provided limited detail regarding annotator training, calibration, or inter-annotator agreement.

Internal structure evidence was reported in only 5 of 13 AI studies (38.5%), direct skill classification models presenting the most consistent evidence, where performance metrics such as accuracy, area under the curve, and cross-validation results were commonly presented. Few studies undertook formal evaluation of reliability or reproducibility across datasets, institutions, or annotators. As such, the extent to which model outputs represent stable and generalisable measures of technical skill remains uncertain.

Generalisability was limited across the majority of studies (8/13 AI studies, 61.5%). Many systems were trained and evaluated on small, single-centre datasets or publicly available video repositories, frequently with incomplete reporting of patient populations, operative difficulty, or case mix. Only one study incorporated a larger multicentre cohort, and few studies assessed performance across different institutions, surgeons, or clinical contexts. Relations to other variables provided limited to moderate evidence of validity. Several studies demonstrated the ability of AI-derived metrics to discriminate between levels of surgical expertise or to correlate with manual-assessment tools and operative duration. However, these associations were inconsistent and largely restricted to surrogate measures of performance, with no evaluation of relationships with patient-level outcomes.

Despite increasing technical sophistication and the emergence of systems capable of real-time intraoperative deployment, no study demonstrated evidence of consequential validity. There was no evaluation of the impact of AI-based assessment on surgical performance, training progression, behavioural modification, or patient safety outcomes such as bile duct injury. Reported benefits were limited to theoretical or anticipated improvements in feedback, standardisation, and intraoperative decision support.

### Methodological quality

Methodological quality, assessed using the MERSQI (maximum score 18), was moderate overall. Across the 60 included studies, total scores ranged from 9.5 to 14.5, with a median of 12.5 (IQR 11.5–13.5). Supplementary Table S5 displays individual study scores.

Most studies employed single-group observational designs and single-centre sampling, resulting in modest scores for study design and sampling domains. In contrast, all studies scored highly for type of data, reflecting use of objective performance metrics. Data analysis approaches were generally appropriate, with most studies reporting reliability statistics, correlation analyses, or algorithm validation metrics. Validity evidence of the evaluation instrument was variably reported, with incomplete psychometric reporting common. Outcomes were limited to performance-level measures in almost all (58/60, 96.7%) of studies; no studies evaluated downstream clinical or patient safety outcomes. Methodological quality was broadly similar across manual, kinematic, and AI-based assessment paradigms, with no clear clustering of higher scores within a specific modality.

### Risk of bias

Risk-of-bias assessments are presented in Supplementary Tables S6–S8, using COSMIN for manual and kinematic studies and QUADAS-2 for AI studies.

Among manual-assessment studies, 22/41 (53.7%) were rated adequate or very good overall by COSMIN, while 11/41 (26.8%) were doubtful and 8/41 (19.5%) inadequate. Methodological quality was generally stronger for reliability and construct-validity analyses, with greater limitations in instrument development and content validity. In contrast, all kinematic studies were rated doubtful or inadequate overall (3/6 [50.0%] each).

Among AI studies, QUADAS-2 identified patient selection as the principal source of potential bias: 4/13 (30.8%) were high risk and 6/13 (46.2%) unclear, whereas risk was low for the index test in 12/13 (92.3%), the reference standard in 11/13 (84.6%), and flow and timing in 12/13 (92.3%). Applicability concerns were uncommon. Overall, 3/13 (23.1%) AI studies were low risk across all domains, 5/13 (38.5%) had at least one high-risk domain, and 5/13 (38.5%) had one or more unclear judgements but no high-risk domain.

## Discussion

### Principal findings

To our knowledge, this is the first systematic review specific to laparoscopic cholecystectomy to evaluate manual, kinematic, and AI-based technical-skill assessment tools within a common Messick validity framework. This should not be interpreted as an absence of such associations across laparoscopic surgery more broadly, where relationships between technical performance and clinical outcomes have previously been reported [8].

Manual-assessment tools are the most well-studied instruments, with the largest body of evidence across multiple independent cohorts. In contrast, kinematic and AI-based systems have predominantly been evaluated within single-centre developmental studies. Across all three paradigms, two common themes emerged. Firstly, tool generalisability was typically weak because of small, selective datasets or single-centre study designs. Secondly, external validity was limited because no included study demonstrated a relationship between tool-derived assessment scores and surgeon behaviour, learning trajectories, or clinical outcomes within laparoscopic cholecystectomy. Without evidence of meaningful clinical benefit, the rationale and value of integrating these tools into routine practice remains uncertain.

### Is there a benchmark or reference standard?

A central aim of this review was to determine whether a gold standard assessment tool exists. No single tool demonstrated comprehensive validity evidence across all Messick domains and therefore, no true gold standard currently exists.

Despite this, some well-established manual tools may have sufficient evidence to act as pragmatic reference standards for emerging technologies, including AI-based systems. Instruments such as GOALS and OSATS have undergone iterative evaluation across multiple institutions and trainee populations, generating a substantial body of evidence. In addition, their widespread familiarity and integration into surgical training enhance their practical utility compared to other tools. However, they cannot be named gold standard tools due to variability in reliability and the absence of consequential or content validity evidence, namely their capacity to alter surgeon behaviours, learning curve trajectories, or clinical outcomes.

In contrast, kinematic and AI-based systems often report apparent strength of internal structure evidence, but this must be interpreted with caution. These are almost wholly single-centre development studies and high performance within a confined dataset does not equate to generalisable construct validity.

Therefore, a clear distinction exists between established tools with accumulated but incomplete validation evidence, and developmental tools with limited and context-specific evidence. This lack of comprehensive, multi-domain validation prevents any current tool from functioning as a true benchmark.

### Readiness for integration into surgical training

The second objective of this review was to evaluate whether any identified tool is ready for integration into surgical training or governance frameworks. At present, no tool demonstrates sufficient evidence to support integration into surgical credentialing or performance monitoring systems.

Manual tools are already embedded in formative training contexts [77], reflecting feasibility and educational familiarity with these tools. However, this does not equate to comprehensive validation. Moreover, these tools rely heavily on trained raters, limiting their scalability, especially for use in continuous monitoring or large-scale implementation.

Kinematic tools demonstrated comparatively weaker validity, partly reflective of the limited number of studies in this category and their narrow construct focus. Although these systems provide objective psychomotor metrics such as instrument velocity, path length, and smoothness, they capture only a limited component of operative competence. They do not assess higher-order skills, such as anatomical judgement, decision-making, or error recognition. Numerous practical barriers also limit their adoption, including sensor deployment, cost, and workflow disruption. Hence, whilst kinematic systems may retain value within research or simulation contexts, their translation into routine intraoperative assessment currently appears limited.

AI-based systems represent the most rapidly expanding domain and demonstrate relatively strong content and response process validity scores, particularly for CVS focussed systems. This likely reflects the international consensus around CVS definitions [78], alongside robust reporting of model performance. These systems offer substantial advantages in efficiency and scalability. Once trained, models can process large volumes of operative footage with minimal human input. Notably, AI-based CVS detection systems are the only tools to demonstrate early real-time intraoperative deployment [41, 48, 51]. However, several concerns continue to limit safe clinical implementation, outlined below [79].

#### Generalisability and dataset representation

Most AI systems were developed and validated using small, single-centre datasets composed predominantly of elective, uncomplicated cases. Severe acute cholecystitis, aberrant anatomy, and inflammatory distortion were severely underrepresented. Deployment beyond the distribution of training data introduces risks related to dataset shift and performance degradation. In high-stakes surgical environments, such miscalibration carries safety implications. Robust generalisability requires heterogeneous, multicentre datasets that reflect the full spectrum of operative complexity.

#### Ground truth integrity

AI performance is contingent on annotation quality [80]. In several studies, annotator expertise, calibration procedures, and inter-rater agreement were incompletely described. Where non-clinical annotators were involved, the reliability of reference standards is uncertain [81]. If labelling processes are opaque or inconsistent, downstream model outputs may lack construct fidelity. Transparent reporting of annotation methodology is therefore essential for reproducibility and safe translation into clinical practice. Importantly, many AI systems rely on manual tools as ground truth during model training, which is of potential consequence as AI outputs may inherit the limitations of the tools used to label them.

#### Alignment between intended use and validity evidence

Many AI systems generate binary classifications (e.g. CVS achieved vs. not achieved). Such outputs may support safety monitoring or threshold-based oversight but provide limited formative value for trainees seeking granular feedback. Conversely, tools intended for credentialing or summative assessment require stronger evidence of reliability and fairness. Evaluation frameworks must therefore align validity evidence with intended application. A mismatch between tool function and evidentiary basis risks inappropriate deployment.

### Implications for safe clinical integration

The introduction of AI systems capable of real-time intraoperative deployment introduces a distinct patient safety and governance challenge. Unlike retrospective manual assessments, real-time algorithmic outputs may directly influence surgeon behaviour, decision-making, and cognitive load within a high-stakes environment. Without structured, staged evaluation, risks include automation bias, overreliance on imperfect signals, and implementation beyond the populations in which systems were validated.

Established frameworks for surgical innovation provide a structured pathway for safe translation. The IDEAL Framework outlines progressive stages of development, exploration, assessment, and long-term study prior to widespread clinical adoption [82, 83]. For AI-based interventions, the DECIDE-AI framework further emphasises prospective evaluation, human–AI interaction assessment, workflow integration, and real-world performance monitoring [84]. Alignment with these staged evaluation models is essential before intraoperative AI systems can be responsibly integrated into surgical training or governance structures. Integration must preserve explicit human oversight and clearly define the role and limits of these AI tools.

### Methodological considerations

Messick’s unified construct-validity framework was selected to enable multidimensional evaluation beyond isolated performance metrics [11]. This approach is particularly important when assessing emerging AI systems, where internal classification accuracy may be robust but broader educational and safety implications remain untested.

A single risk-of-bias tool was not appropriate for all included studies because the studies addressed fundamentally different questions. Manual and kinematic studies primarily examined how well technical-skill assessment tools performed, for example whether they produced reliable scores and whether they could distinguish differences in surgical performance. These studies were therefore assessed using COSMIN. In contrast, AI studies mainly examined how accurately an algorithm’s assessment agreed with an expert-derived or annotated reference standard and were therefore assessed using QUADAS-2. This allowed methodological quality to be assessed using criteria appropriate to the type of evidence generated by each study, rather than applying a framework developed for studies of intervention effects.

Methodological quality differed substantially by modality. Over half of manual-assessment studies were rated adequate or very good, whereas all kinematic studies were rated doubtful or inadequate, indicating that the use of quantitative motion-based metrics did not necessarily correspond to stronger validation methodology. In AI studies, patient selection was the principal risk-of-bias concern, while other QUADAS-2 domains were generally low risk. Overall, this raises particular concerns regarding generalisability, as performance derived from selected or poorly characterised datasets may not translate to broader operative populations or settings. These findings support the need for prospective validation in representative clinical cohorts before such tools are used for credentialling or performance monitoring.

### Limitations

Heterogeneity in study design, populations, constructs assessed, and reported outcomes precluded quantitative meta-analysis. The review was restricted to clinical laparoscopic cholecystectomy, excluding simulation-based and pre-clinical studies, which may underestimate earlier-stage innovation. Additionally, we did not include studies examining non-technical skills assessments, which are arguably of equal importance in the operating room [85]. Publication bias cannot be excluded, particularly within rapidly expanding AI literature. Additionally, aggregation of Messick validity ratings at the tool level may favour tools evaluated in single studies, as their performance has not been challenged across multiple cohorts or settings.

Finally, deviations from the pre-registered protocol included omission of OCEBM and GRADE-based assessments and replacement of ROBINS-I with COSMIN and QUADAS-2 for risk-of-bias assessment. ROBINS-I was not considered appropriate because the included studies predominantly evaluated assessment-tool validity or algorithmic accuracy rather than non-randomised intervention effects; the rationale for using COSMIN and QUADAS-2 is described above. OCEBM and GRADE were also not applied because the review did not synthesise a common intervention effect or other single body of evidence for which an overall certainty rating could be meaningfully assigned. Messick’s validity framework, complemented by MERSQI, provided a more appropriate structure for evaluating assessment tools in this context.

### Future research agenda

To transition from developmental validation to safe clinical integration of objective technical skills assessment tools, future research should:

1. Conduct prospective, multicentre validation across heterogeneous pathology and surgeon experience levels.
2. Establish transparent and standardised annotation protocols to strengthen ground truth reliability.
3. Evaluate behavioural changes, learning curves, and patient outcomes, rather than relying solely on technical classification metrics.
4. Develop shared benchmark datasets for laparoscopic cholecystectomy to enable reproducible external validation.
5. Explicitly align tool design with intended use, distinguishing between formative feedback, monitoring, and credentialing functions.

## Conclusion

Manual tools remain the most established modality for technical skills assessment but are constrained by variability and limited scalability. Kinematic systems provide objective metrics yet capture only a narrow dimension of operative competence. AI-based video systems demonstrate significant technical promise, particularly in safety–critical construct detection, and offer the possibility of scalable intraoperative monitoring.

No single tool demonstrates comprehensive validity evidence across all Messick domains and therefore, no true gold standard currently exists. Across all paradigms, generalisability is limited and there is minimal evidence that these tools improve surgical performance or patient outcomes. No current tool meets the requirements for integration into surgical credentialing or performance monitoring systems.

Future progress will depend not on further technical refinement alone, but on demonstrating meaningful impact on surgical performance and patient outcomes.

## Supplementary Information

Below is the link to the electronic supplementary material.

Supplementary file1 (DOCX 269 kb)

Supplementary file2 (DOCX 20 kb)

Supplementary file3 (DOCX 26 kb)

Supplementary file4 (CSV 28 kb)

Supplementary file5 (DOCX 17 kb)

Supplementary file6 (DOCX 23 kb)

Supplementary file7 (DOCX 35 kb)

Supplementary file8 (DOCX 27 kb)

Supplementary file9 (DOCX 17 kb)

Supplementary file9 (DOCX 223 kb)

## Data Availability

All data analysed in this study are derived from previously published articles, which are cited within the manuscript. The data extraction forms and datasets generated during the current study are available from the corresponding author upon reasonable request.

## Declarations

### Previous communications

This manuscript has not been previously presented at any scientific meeting or conference and is not under consideration for publication elsewhere.
