# Supplementary material for: Objective technical skills assessment in laparoscopic cholecystectomy: a systematic review of manual, kinematic, and AI-based tools
Source: Surg Endosc. 2026 Aug 31;40(10):8285–303. doi: 10.1007/s00464-026-13309-z (PMC13633379; doi:10.1007/s00464-026-13309-z)
Supplement: Supplementary file 1 [file 464_2026_13309_MOESM10_ESM.docx]

**Supplementary methods: full search strategy**

**Databases**: Embase, Medline, Web of Science, Cochrane library

**Dates of search**: from inception to 12^th^ of August 2025

**Search terms:**

**MEDLINE (Ovid)**
Searched from inception to 12 August 2025.

1. Laparoscopic cholecystectom*.ti,ab,kf.
2. laparoscopic cholecystectomy/
3. 1 or 2
4. clinical competence/ or skill/ or competency based medical education/ or competency-based education/ or professional competence/
5. (skill* or performance or competen* or proficie* or expert* or capabilit*).ti,ab,kf.
6. 4 or 5
7. scoring system/ or "performance measurement system"/
8. (valid* or evaluat* or assess* or objective or formative or summative or analys* or score* or scoring or measure* or performance* or instrument or instruments or indicies or index* or scale or scales or screen or screened or screening or surve* or checklist* or questionnaire* or tool or tools or observation forms or tally sheets* or OSATS or HRA or "human reliability analysis" or GOALS or OCHRA or error* or feedback).ti,ab,kf.
9. 7 or 8
10. 3 and 6 and 9

**Records retrieved on 12 August 2025: 1,505**

**Embase (Ovid)**
Searched from inception to 12 August 2025.

1. "Laparoscopic cholecystectom*".tw,kf.
2. "laparoscopic cholecystectomy"/
3. 1 or 2
4. "clinical competence"/ or skill/ or "competency based medical education"/ or "competency-based education"/ or "professional competence"/
5. (skill* or performance or competen* or proficie* or expert* or capabilit*).tw,kf.
6. 4 or 5
7. scoring system/ or "performance measurement system"/
8. (valid* or evaluat* or assess* or objective or formative or summative or analys* or score* or scoring or measure* or performance* or instrument or instruments or indicies or index* or scale or scales or screen or screened or screening or surve* or checklist* or questionnaire* or tool or tools or "observation forms" or "tally sheets*" or OSATS or HRA or "human reliability analysis" or GOALS or OCHRA or error* or feedback).tw,kf.
9. 7 or 8
10. 3 and 6 and 9

**Records retrieved on 12 August 2025: 2,195**

**Web of Science**

Searched from inception to 12 August 2025.

TS=("Laparoscopic cholecystectom*" OR "laparoscopic cholecystectomy")

AND

TS=("clinical competence" OR "skill" OR "competency based medical education" OR "competency-based education" OR "professional competence" OR "skill*" OR "performance" OR "competen*" OR "proficie*" OR "expert*" OR "capabilit*")

AND

TS=("scoring system" OR "performance measurement system" OR "valid*" OR "evaluat*" OR "assess*" OR "objective" OR "formative" OR "summative" OR "analys*" OR "score*" OR "scoring" OR "measure*" OR "performance*" OR "instrument" OR "instruments" OR "indices" OR "index*" OR "scale" OR "scales" OR "screen" OR "screened" OR "screening" OR "surve*" OR "checklist*" OR "questionnaire*" OR "tool" OR "tools" OR "observation forms" OR "tally sheets*" OR "OSATS" OR "HRA" OR "human reliability analysis" OR "GOALS" OR "OCHRA" OR "error*" OR "feedback")

**Records retrieved on 12 August 2025: 2073**

**Cochrane Library**

Searched from inception to 12 August 2025.

#1	("Laparoscopic" NEXT cholecystectom*):ti,ab,kw	5866

#2	[mh ^"laparoscopic cholecystectomy"]	1787

#3	#1 OR #2	5999

#4	[mh ^"clinical competence"] OR [mh ^skill] OR [mh ^"competency based medical education"] OR [mh ^"competency-based education"] OR [mh ^"professional competence"]	5773

#5	(skill*:ti,ab,kw OR performance:ti,ab,kw OR competen*:ti,ab,kw OR proficie*:ti,ab,kw OR expert*:ti,ab,kw OR capabilit*:ti,ab,kw)	204568

#6	#4 OR #5	204568

#7	[mh ^"scoring system"] OR [mh ^"performance measurement system"]	0

#8	(valid*:ti,ab,kw OR evaluat*:ti,ab,kw OR assess*:ti,ab,kw OR objective:ti,ab,kw OR formative:ti,ab,kw OR summative:ti,ab,kw OR analys*:ti,ab,kw OR score*:ti,ab,kw OR scoring:ti,ab,kw OR measure*:ti,ab,kw OR performance*:ti,ab,kw OR instrument:ti,ab,kw OR instruments:ti,ab,kw OR indicies:ti,ab,kw OR index*:ti,ab,kw OR scale:ti,ab,kw OR scales:ti,ab,kw OR screen:ti,ab,kw OR screened:ti,ab,kw OR screening:ti,ab,kw OR surve*:ti,ab,kw OR checklist*:ti,ab,kw OR questionnaire*:ti,ab,kw OR tool:ti,ab,kw OR tools:ti,ab,kw OR "observation forms":ti,ab,kw OR ("tally" NEXT sheets*):ti,ab,kw OR OSATS:ti,ab,kw OR HRA:ti,ab,kw OR "human reliability analysis":ti,ab,kw OR GOALS:ti,ab,kw OR OCHRA:ti,ab,kw OR error*:ti,ab,kw OR feedback:ti,ab,kw)	1723182

#9	#7 OR #8	1723182

#10	#3 AND #6 AND #9	327

**Records retrieved on 12 August 2025: 325**
