# Supplementary material for: Objective technical skills assessment in laparoscopic cholecystectomy: a systematic review of manual, kinematic, and AI-based tools
Source: Surg Endosc. 2026 Aug 31;40(10):8285–303. doi: 10.1007/s00464-026-13309-z (PMC13633379; doi:10.1007/s00464-026-13309-z)
Supplement: Supplementary file 3 [file 464_2026_13309_MOESM2_ESM.docx]

**Supplementary Table S1. Messick’s validity framework and evidence rating rubric used to appraise technical skills assessment tools for laparoscopic cholecystectomy.** For each validity domain, the table provides the working definition, descriptive rating criteria, and illustrative examples used in this review. Validity evidence was classified as not reported, limited, partial, or strong according to the extent and quality of information available within each study. The rubric was adapted by the authors from Messick’s unified validity framework for application to studies of operative performance assessment

| **Domain** | **Definition** | **Evidence Rating** (numeric score) | **Description** | **Examples** |
| --- | --- | --- | --- | --- |
| Content validity | The extent to which the tool’s content is relevant and represents the construct it intends to measure. | Not reported (–) | No data regarding the content validity |  |
|  |  | Limited (1) | Expert judgment with limited data regarding the tool content | Two experts have devised a tool based on their opinions |
|  |  | Partial (2) | Listing assessment items for the tool content with some references to a panel of experts, limited description of the developing process  References to a previously validated tool | Structured task analysis, hierarchical task analysis  Based on previously validated tools |
|  |  | Strong (3) | Well-defined developing process, both theoretical basis for the chosen items and systematic review by experts | Delphi method  Or  Expert-derived and universally recognised framework for defining safe dissection during LC. |
| Response process | The extent to which the **processes used to generate assessment scores** (by human raters, learners, or AI systems) are consistent with the intended construct being measured, and are applied **accurately, fairly, and without bias. processes underlying performance on the construct being measured.** | Not reported (–) | No data regarding the response process |  |
|  |  | Limited (1) | Limited data reported. Use of an assessment tool without discussing the impact of the differences in response processes | Assessors only given user manuals or equivalent |
|  |  | Partial (2) | Some data regarding different responses of assessors. Some data about systems that reduce variation between respondents | Assessors given structured assessor training before the assessment process |
|  |  | Strong (3) | Multiple sources of data examining response error through critical examination of response processes and respondents | Assessors trained and then tested on their scores, to ensure that they are sufficiently calibrated, prior to being let loose on the study population. |
| Internal structure | The extent to which individual items describe the underlying constructs, often reported by measures of inter-rater reliability, internal consistency and generalizability | Not reported (–) | No data regarding the internal structure |  |
|  |  | Limited (1) | Weak or inadequate evidence of reliability | Low inter-rater reliability coefficients; poorly described or unclear reliability methods |
|  |  | Partial (2) | A few measures of reliability reported, insufficiently item analysis  Adequate inter-rater reliability demonstrated, with or without additional measures of internal consistency | Moderate–high inter-rater reliability coefficients (e.g. ICC); inter-rater reliability combined with Cronbach’s alpha or inter-item reliability |
|  |  | Strong (3) | Multiple robust analyses of internal structure demonstrating consistency across items and raters | Generalisability theory analysis; inter-rater reliability combined with advanced methods such as factor analysis or item response theory |
| Generalisability | The extent to which the tool’s results apply across different learners, institutions, case types, and contexts. | Not reported (–) | No data regarding generalisability. | Tool tested only in a single institution with one group of surgeons, no description of learner mix or case variation. |
|  |  | Limited (1) | Limited data: tested in a single site with some variation in learner group or case type. | Checklist tested only at one hospital but includes both residents and consultants. AI tool trained/tested on elective cases only. |
|  |  | Partial (2) | Some generalisability data: tested across broader learner groups or more than one site, but without external validation. | Tool applied at 2 hospitals or across novices, intermediates, and experts; no external dataset for AI. |
|  |  | Strong (3) | Strong evidence: tested across multiple institutions, learner levels, and/or externally validated on independent data. | AI model trained at Hospital A, then validated on independent datasets from Hospitals B and C, including novices and experts, elective and emergency cases, and variable difficulty. Checklist validated across >3 institutions internationally. |
| Relations to other variables | Correlation between assessment scores and other outcomes or scoring systems relevant to the construct being measured | Not reported (–) | No data regarding the other variables |  |
|  |  | Limited (1) | Correlation of assessment scores with experience or another tool | Tool validated by experience or another tool |
|  |  | Partial (2) | Correlation of assessment scores with experience and another tool | Tool validated by experience **and** another tool |
|  |  | Strong (3) | Correlation between assessment scores and clinical outcomes | Tool validated by clinical outcomes |
| Consequences | The impact of the assessment and future use (both intended and unintended) | Not reported (–) | No data regarding the consequences |  |
|  |  | Limited (1) | Limited data, merely a discussion about future use | Describing feasibility and potential future use (data on assessment time, post assessment survey) |
|  |  | Partial (2) | Some descriptions of consequences of assessment for learners, often supported by incomplete data | Describing educational impact (formative/summative feedback, learning curve of trainees) |
|  |  | Strong (3) | Clear description of consequences of assessments and the impact on interpretation of scores and intended future use, supported by data | Criterion-referenced score (pass/fail-scores), cut-off scores for licensing purposes, predictive models |
