# Supplementary material for: Objective technical skills assessment in laparoscopic cholecystectomy: a systematic review of manual, kinematic, and AI-based tools
Source: Surg Endosc. 2026 Aug 31;40(10):8285–303. doi: 10.1007/s00464-026-13309-z (PMC13633379; doi:10.1007/s00464-026-13309-z)
Supplement: Supplementary file 4 [file 464_2026_13309_MOESM3_ESM.docx]

**Supplementary Table S2**. **Full-text articles excluded after eligibility assessment, with reasons for exclusion**.

| Study | Title | Reason for exclusion | DOI |
| --- | --- | --- | --- |
| Balvardi 2022 | Validity of video-based generic and procedure-specific self-assessment tools for surgical trainees in laparoscopic cholecystectomy | Conference abstract only | https://dx.doi.org/10.1007/s00464-022-09218-6 |
| Bilgic 2014 | Reliability of goals scores using generalizability theory | Conference abstract only | https://dx.doi.org/10.1007/s00464-014-3476-z |
| Bonomo 2012 | Multi-centre study on operative performance in minimal access surgery | Conference abstract only | https://dx.doi.org/10.1007/s00464-014-3484-z |
| Campioni-Norman 2024 | Reliability of Simulation-Based Assessment of Surgical Technical Skills | Conference abstract only | https://dx.doi.org/10.1093/bjs/znae163.069 |
| Chen 2022 | Surgesture: A Novel Instrument Based on Surgical Actions for Objective Skill Assessment | Conference abstract only | https://dx.doi.org/10.1016/j.hpb.2022.05.1167 |
| Chevallay 2019 | Competence in laparoscopic cholecystectomy, implementation of an assessment tool in the fellowship program | Conference abstract only | https://dx.doi.org/10.1007/s00464-019-06704-2 |
| CifrianCanales 2021 | Implementation of the evaluation to improve resident surgical skills | Conference abstract only | https://dx.doi.org/10.1093/bjs/znab160.065 |
| Deal 2017 | An overwhelming majority of public domain, surgical videos of laparoscopic cholecystectomy do not demonstrate the critical view of safety | Conference abstract only | https://dx.doi.org/10.1016/j.hpb.2017.02.084 |
| Deal 2017 | Concordance between crowd workers and faculty experts in assessment of the critical view of safety in laparoscopic cholecystectomy using crowd-sourced assessment of technical skills | Conference abstract only | https://dx.doi.org/10.1007/s00464-017-5446-8 |
| Deal 2017 | Technical performance does not correlate with completion of the critical view of safety as assessed by crowd worker and faculty expert evaluations of laparoscopic cholecystectomy | Conference abstract only | https://dx.doi.org/10.1007/s00464-017-5444-x |
| Hegde 2022 | Automated Prediction of Critical View of Safety Criteria in Laparoscopic Cholecystectomy Using Deep Learning | Conference abstract only | https://dx.doi.org/10.1097/01.XCS.0000893480.53682.a4 |
| Jung 2014 | Laparoscopic Competency Assessment Tool (LCAT) for technical surgical performance in laparoscopic cholecystectomy at the resident level | Conference abstract only |  |
| Kakucs 2016 | Laparoscopic skill assessment using not selected unedited blinded videos of laparoscopic cholecystectomies | Conference abstract only | https://dx.doi.org/10.1007/s00464-016-4766-4 |
| Kramp 2015 | Goals assessment of three key steps in laparoscopic cholecystectomy | Conference abstract only | https://dx.doi.org/10.1007/s00464-015-4136-7 |
| Lavanchy 2021 | Surgical skill assessment using machine learning algorithms | Conference abstract only | https://dx.doi.org/10.1093/bjs/znab202.093 |
| Lukovich 2015 | The quality control of laparoscopic technique | Conference abstract only | https://dx.doi.org/10.1007/s00464-015-4135-8 |
| Mahroos 2018 | Development and validity evidence for a new comprehensive intra-operative assessment tool: A pilot study | Conference abstract only | https://dx.doi.org/10.1007/s00464-018-6121-4 |
| Martin 2018 | Novel Competency-Based Curriculum for Laparoscopic Cholecystectomy Decreases Operative Performance Variability among Surgery Residents | Conference abstract only | https://dx.doi.org/10.1016/j.jamcollsurg.2018.07.489 |
| Matsumoto 2012 | The quality of endoscopic surgical skill qualification in Japan in the section of laparoscopic cholecystectomy | Conference abstract only | https://dx.doi.org/10.1007/s00464-012-2203-x |
| Namazi 2020 | AI for Automated Detection of the Establishment of Critical View of Safety in Laparoscopic Cholecystectomy Videos | Conference abstract only | https://dx.doi.org/10.1016/j.jamcollsurg.2020.08.118 |
| Namazi 2021 | Automatic Hierarchical Task Segmentation of Laparoscopic Cholecystectomy Videos Using Deep Learning | Conference abstract only | https://dx.doi.org/10.1007/s00464-021-08747-w |
| Nasim 2016 | Prospective Video-Based Assessment of Trainee Performance during Laparoscopic Cholecystectomy; Utility of a Mayo Intraoperative 10-Step Check List | Conference abstract only |  |
| PintoAngulo 2014 | The routine application system Observational Human Clinical Assessment Reliability (OCHRA) gradually decreases errors during laparoscopic cholecystectomy | Conference abstract only | https://dx.doi.org/10.1016/j.jamcollsurg.2014.07.819 |
| Sobba 2018 | Live Quality Assurance: Using a Short T Message Service Group Chat to Instantly Grade Intraoperative Images | Conference abstract only | https://dx.doi.org/10.1016/j.jamcollsurg.2018.07.214 |
| Sobba 2019 | Live QA, live image sharing and grading to evaluate the CVS in cholecystitis | Conference abstract only | https://dx.doi.org/10.1007/s00464-019-06704-2 |
| Soltani 2023 | Thematic Analysis of Narrative Feedback in the System for Improving and Measuring Procedural Learning Platform | Conference abstract only | https://dx.doi.org/10.1097/XCS.0000000000000839 |
| Szasz 2016 | Setting Performance Standards for Technical and Nontechnical Competence in General Surgery | Conference abstract only | https://dx.doi.org/10.1016/j.jamcollsurg.2016.06.277 |
| Szasz 2016 | Subjective and Objective Performance Assessments Correlate in the Operating Room | Conference abstract only | https://dx.doi.org/10.1016/j.jamcollsurg.2016.06.280 |
| Tan 2023 | Effect of Artificial Intelligence Mobile Application Software as a Guide in Identifying the Critical View of Safety in Laparoscopic Cholecystectomy: A Pilot Randomized Control Trial | Conference abstract only | https://dx.doi.org/10.1097/XCS.0000000000000831 |
| Villarreal 2024 | The Future Is in Sight-Using Computer Vision for Automated Objective Review of Laparoscopic Cholecystectomies | Conference abstract only | https://dx.doi.org/10.1097/XCS.0000000000001177 |
| Wallace 2017 | Surgical chief resident competency in laparoscopic and robotic surgery: Are we ready? | Conference abstract only | https://dx.doi.org/10.1007/s00464-017-5445-9 |
| Balvardi 2024 | Effect of video-based self-reflection on intraoperative skills: A pilot randomized controlled trial. | Wrong outcome | https://dx.doi.org/10.1016/j.surg.2023.11.028 |
| Deal 2017 | Concerns of Quality and Safety in Public Domain Surgical Education Videos: An Assessment of the Critical View of Safety in Frequently Used Laparoscopic Cholecystectomy Videos. | Wrong outcome | https://dx.doi.org/10.1016/j.jamcollsurg.2017.08.016 |
| Deal 2017 | Evaluation of crowd-sourced assessment of the critical view of safety in laparoscopic cholecystectomy. | Wrong outcome | https://dx.doi.org/10.1007/s00464-017-5574-1 |
| ElBoghdady 2016 | The effect of surgical checklists on the laparoscopic task performance | Wrong outcome |  |
| ElBoghdady 2018 | The Application of a Performance-Based Self-Administered Intra-procedural Checklist on Surgical Trainees During Laparoscopic Cholecystectomy. | Wrong outcome | https://dx.doi.org/10.1007/s00268-017-4361-4 |
| Evans-Harvey 2020 | Comparison of surgeon gaze behaviour against objective skill assessment in laparoscopic cholecystectomy-a prospective cohort study. | Wrong outcome | https://dx.doi.org/10.1016/j.ijsu.2020.08.006 |
| Faber 2023 | Implementation of an Online Intraoperative Assessment of Technical Performance for Surgical Trainees | Wrong outcome | https://dx.doi.org/10.1016/j.jss.2023.07.008 |
| Fried 2024 | Surgical Intelligence Can Lead to Higher Adoption of Best Practices in Minimally Invasive Surgery. | Wrong outcome | https://dx.doi.org/10.1097/SLA.0000000000006377 |
| Fryer 2018 | Effect of Ongoing Assessment of Resident Operative Autonomy on the Operating Room Environment | Wrong outcome | https://dx.doi.org/10.1016/j.jsurg.2016.11.018 |
| Gruter 2024 | Surgical quality assessment of critical view of safety in 283 laparoscopic cholecystectomy videos by surgical residents and surgeons. | Wrong outcome | https://dx.doi.org/10.1007/s00464-024-10873-0 |
| Guerlain 2005 | Assessing team performance in the operating room: development and use of a "black-box" recorder and other tools for the intraoperative environment. | Wrong outcome | https://dx.doi.org/10.1016/j.jamcollsurg.2004.08.029 |
| Hegde 2024 | Automated segmentation of phases, steps, and tasks in laparoscopic cholecystectomy using deep learning. | Wrong outcome | https://dx.doi.org/10.1007/s00464-023-10482-3 |
| IEEE 2019 | The Effect of Expertise on Gaze Behaviour in Laparoscopic Cholecystectomy | Wrong outcome | 10.1109/embc.2019.8857612 |
| Jackson 2021 | ATTENDING GUIDANCE ADVISED: EDUCATIONAL QUALITY of SURGICAL VIDEOS on YOUTUBE | Wrong outcome | https://dx.doi.org/10.1007/s00464-021-08747-w |
| Jin 2022 | <SUP>???????</SUP>Critical view of safety in laparoscopic cholecystectomy: A prospective investigation from both cognitive and executive aspects | Wrong outcome | 10.3389/fsurg.2022.946917 |
| Meholick 2020 | Use of a Secure Web-Based Data Management Platform to Track Resident Operative Performance and Program Educational Quality Over Time | Wrong outcome | https://dx.doi.org/10.1016/j.jsurg.2020.05.022 |
| Quick 2017 | Objective Measurement of Clinical Competency in Surgical Education Using Electrodermal Activity. | Wrong outcome | https://dx.doi.org/10.1016/j.jsurg.2017.01.007 |
| Sanfey 2011 | Evaluating resident operative performance: a qualitative analysis of expert opinions. | Wrong outcome | https://dx.doi.org/10.1016/j.surg.2011.07.058 |
| Stefanidis 2017 | How often do surgeons obtain the critical view of safety during laparoscopic cholecystectomy?. | Wrong outcome | https://dx.doi.org/10.1007/s00464-016-4943-5 |
| Surg Endosc 23:1476–1482 | Surg Endosc 23:1476–1482 | Wrong outcome |  |
| Tranter-Entwistle 2024 | Bayesian process mapping for intraoperative decision making in laparoscopic cholecystectomy | Wrong outcome | 10.20517/ais.2024.04 |
| Williams 2019 | How Many Observations are Needed to Assess a Surgical Trainee's State of Operative Competency?. | Wrong outcome | https://dx.doi.org/10.1097/SLA.0000000000002554 |
| Yildirim 2021 | Performance of Residents During Laparoscopic Cholecystectomy: Is Self-assessment Reliable?. | Wrong outcome | https://dx.doi.org/10.1097/SLE.0000000000000959 |
| Adrales 2023 | A Multi-national video-Based Qualitative Study to Refine Training Guidelines for Assigning an "Unsafe" Score in Laparoscopic Cholecystectomy Critical View of Safety | Wrong intervention | https://dx.doi.org/10.1007/s00464-023-10071-4 |
| Athanasiadis 2025 | How Accurate Are Surgeons at Assessing the Quality of Their Critical View of Safety During Laparoscopic Cholecystectomy?. | Wrong intervention | https://dx.doi.org/10.1016/j.jss.2024.10.048 |
| Byrne 2000 | Telementoring as an adjunct to training and competence-based assessment in laparoscopic cholecystectomy. | Wrong intervention | https://dx.doi.org/10.1007/s004640000264 |
| Effect of Video-based Guided Self-reflec | Effect of Video-based Guided Self-reflection on Intraoperative Skills: a Pilot Randomized Controlled TrialA1 - Anonymous. | Wrong intervention |  |
| Reitano 2023 | Educational Scoring System in Laparoscopic Cholecystectomy: Is It the Right Time to Standardize?. | Wrong intervention | https://dx.doi.org/10.3390/medicina59030446 |
| Validation of a Structured Training and | Validation of a Structured Training and Assessment Curriculum (STAC) for Technical Skill Acquisition in Minimally Invasive Surgery. A Randomized Controlled StudyA1 - Anonymous. | Wrong intervention |  |
| Adrales 2023 | Laparoscopic Cholecystectomy Critical View of Safety (LC-CVS): A Multi-national Validation Study of an Objective, Procedure- Specific Assessment Using Video-Based Assessment (VBA) | Wrong study design | https://dx.doi.org/10.1007/s00464-023-10071-4 |
| AlAbbas 2023 | The Implementation of a Novel Artificial Intelligence Platform for Resident Assessment on an Emergency General Surgery Service: An Operating Room Black Box Analysis | Wrong study design | https://dx.doi.org/10.1097/XCS.0000000000000839 |
| Dick 2024 | Automated analysis of operative video in surgical training: scoping review. | Wrong study design | https://dx.doi.org/10.1093/bjsopen/zrae124 |
| Huang 2018 | Video-based performance assessment in endoscopy: Moving beyond "see one, do one, teach one"? | Wrong study design | https://dx.doi.org/10.1016/j.gie.2017.09.014 |
| Rios 2023 | Cholec80-CVS: An open dataset with an evaluation of Strasberg's critical view of safety for AI. | Wrong study design | https://dx.doi.org/10.1038/s41597-023-02073-7 |
| Ryan 2022 | Validity Evidence for Procedure-specific Competence Assessment Tools in General Surgery: A Scoping Review | Wrong study design | https://dx.doi.org/10.1097/SLA.0000000000005207 |
| Sarker 2007 | Assessment of technical skills in surgery | Wrong study design |  |
| Watanabe 2016 | A systematic review of performance assessment tools for laparoscopic cholecystectomy. | Wrong study design | https://dx.doi.org/10.1007/s00464-015-4285-8 |
| Alibhai 2022 | Assessment of laparoscopic skills: comparing the reliability of global rating and entrustability tools. | Wrong patient population | https://dx.doi.org/10.36834/cmej.72369 |
| Ganni 2017 | Self-assessment in laparoscopic surgical skills training: Is it reliable?. | Wrong patient population | https://dx.doi.org/10.1007/s00464-016-5246-6 |
| Gawad 2019 | The Inter-Rater Reliability of Technical Skills Assessment and Retention of Rater Training. | Wrong patient population | https://dx.doi.org/10.1016/j.jsurg.2019.01.001 |
| Shabahang 2022 | American College of Surgeons Objective Assessment of Skills in Surgery (ACS OASIS): A Formative Assessment of Junior Residents' Technical Skills. | Wrong setting | https://dx.doi.org/10.1016/j.jsurg.2022.07.007 |
| Tang 2006 | Competence assessment of laparoscopic operative and cognitive skills: Objective Structured Clinical Examination (OSCE) or Observational Clinical Human Reliability Assessment (OCHRA). | Wrong setting | https://dx.doi.org/10.1007/s00268-005-0157-z |
| denBoer 2001 | Surgical task performance: Assessment using time -action analysis | Full text not available in English |  |
| Kang 2020 | Objective differences in technical performance during laparoscopic cholecystectomy between SET Trainees and Surgeons using video benchmarking | Full text not available in English | https://dx.doi.org/10.1002/bjs.12067 |
| Kasparian 2014 | [Evaluation of technical skills in surgical training]. | Full text not available in English |  |
| McBeth 2002 | Quantitative methodology of evaluating surgeon performance in laparoscopic surgery. | Full text not available in English |  |
| NCT04643314 2020 | Effect of Video-based Guided Self-reflection on Intraoperative Skills | Full text not available in English |  |
| Neeraj 2013 | Monitoring Quality of Laparoscopic Cholecystectomy by Analysis of Critical View of Safety | Full text not available in English |  |
| Zhang 2024 | LC-Smart: A Multicentric Deep Learning-Based Quality Control Model for Laparoscopic Cholecystectomy | Full text not available in English | https://dx.doi.org/10.2139/ssrn.5058464 |
