# Supplementary material for: Objective technical skills assessment in laparoscopic cholecystectomy: a systematic review of manual, kinematic, and AI-based tools
Source: Surg Endosc. 2026 Aug 31;40(10):8285–303. doi: 10.1007/s00464-026-13309-z (PMC13633379; doi:10.1007/s00464-026-13309-z)
Supplement: Supplementary file 5 [file 464_2026_13309_MOESM5_ESM.docx]

**Supplementary Table S4. Sources of clinical and methodological heterogeneity across assessment modalities and implications for quantitative synthesis.** Characteristics are summarised across manual, kinematic, and AI-based technical skills assessment studies according to study design, population, construct assessed, clinical setting, assessment method, reference or comparator, and reported outcomes. Differences across these domains limited the comparability of effect estimates and precluded clinically and methodologically meaningful quantitative pooling.

| **Source of heterogeneity** | **Manual assessment studies** | **Kinematic studies** | **AI-based studies** | **Implication for quantitative synthesis** |
| --- | --- | --- | --- | --- |
| **Construct assessed** | Global technical skill, procedure-specific performance, errors, CVS attainment | Economy of movement, path length, time, instrument use | Skill classification, phase recognition, CVS detection, segmentation | Different aspects of technical performance were assessed, limiting comparability across studies. |
| **Study design** | Predominantly retrospective observational studies, with some prospective studies and one randomised controlled trial | Mixed prospective intraoperative data capture with retrospective analysis, and retrospective observational validation studies | Retrospective model-development and validation studies, prospective observational studies, and real-time evaluation | Differences in study design and analytical approach limit comparability across studies |
| **Population** | Novices, trainees and experts, variably defined | Novices, trainees and experts, variably defined | Mixed operators and heterogeneous video datasets | No consistent population or expertise threshold from which to derive a common effect |
| **Clinical setting** | Elective LC, emergency LC or mixed cases | Elective LC, or mixed cases | Retrospective video datasets and prospective intraoperative application, with variable case selection and operative complexity | Substantial differences in case spectrum, operative complexity and spectrum of difficulty |
| **Assessment instrument** | Multiple rating scales and procedure-specific tools with different domains and scoring ranges | Different sensors, tracking systems and derived kinematic metrics | Different model architectures, inputs and target labels | Scores and metrics were not directly comparable |
| **Reference/comparator** | Expert opinion, experience level, other rating instruments or clinical criteria | Experience group or manual assessment score | Expert annotation, or consensus labels | No consistent reference standard was used across studies |
| **Reported outcomes** | ICC, kappa, correlations, between-group differences and validity evidence | Correlations, group discrimination and classification performance | Accuracy, sensitivity, specificity, AUC, F1, IoU | No common outcome measure was available for meaningful statistical pooling. |

AI = artificial intelligence; LC = laparoscopic cholecystectomy; CVS = critical view of safety; ICC = intraclass correlation coefficient; AUC = area under the curve; F1 = F1 score; IoU = intersection over union.
