# Supplementary material for: Objective technical skills assessment in laparoscopic cholecystectomy: a systematic review of manual, kinematic, and AI-based tools
Source: Surg Endosc. 2026 Aug 31;40(10):8285–303. doi: 10.1007/s00464-026-13309-z (PMC13633379; doi:10.1007/s00464-026-13309-z)
Supplement: Supplementary file 6 [file 464_2026_13309_MOESM6_ESM.docx]

**Supplementary table S5. Study-level methodological quality appraisal using the Medical Education Research Study Quality Instrument (MERSQI).** For each included study, the table reports domain-level MERSQI scores for study design, sampling, type of data, validity of the evaluation instrument, data analysis, and outcomes, together with the total score. Higher scores reflect stronger methodological quality.

| **Paper** | **Publication date** | **Study Design** | **Sampling** | **Types of Data** | **Validity of Evaluation Instrument** | **Data Analysis** | **Outcomes** | **TOTAL** |
| --- | --- | --- | --- | --- | --- | --- | --- | --- |
| Wu et al, | 2023 | 1 | 3 | 3 | 3 | 3 | 1.5 | 14.5 |
| Chen et al, | 2024 | 1 | 2 | 3 | 3 | 3 | 1.5 | 13.5 |
| Eubanks et al, | 1999 | 1 | 2 | 3 | 3 | 3 | 1.5 | 13.5 |
| Hopmans et al, | 2014 | 1 | 2 | 3 | 3 | 3 | 1.5 | 13.5 |
| Humm et al, | 2023 | 1 | 2 | 3 | 3 | 3 | 1.5 | 13.5 |
| Lam et al, | 2014 | 1 | 2 | 3 | 3 | 3 | 1.5 | 13.5 |
| Malik et al, | 2016 | 1 | 2 | 3 | 3 | 3 | 1.5 | 13.5 |
| Sarker et al, | 2010 | 1 | 2 | 3 | 3 | 3 | 1.5 | 13.5 |
| Szasz et al, | 2017a | 1 | 2 | 3 | 3 | 3 | 1.5 | 13.5 |
| Vassiliou et al, | 2005 | 1 | 2 | 3 | 3 | 3 | 1.5 | 13.5 |
| Sarker et al, | 2006c | 1 | 1.5 | 3 | 3 | 3 | 1.5 | 13 |
| Sarker et al, | 2006b | 1 | 1.5 | 3 | 3 | 3 | 1.5 | 13 |
| Vassiliou et al, | 2007 | 1 | 1.5 | 3 | 3 | 3 | 1.5 | 13 |
| Yanik et al, | 2024 | 1 | 1.5 | 3 | 3 | 3 | 1.5 | 13 |
| Chang et al, | 2007 | 1 | 1 | 3 | 3 | 3 | 1.5 | 12.5 |
| Cristancho et al, | 2008 | 1 | 1 | 3 | 3 | 3 | 1.5 | 12.5 |
| Huber et al, | 2018 | 1 | 1 | 3 | 3 | 3 | 1.5 | 12.5 |
| Humm et al, | 2023 | 1 | 1 | 3 | 3 | 3 | 1.5 | 12.5 |
| Kavuluri et al, | 2025 | 3 | 1 | 3 | 1 | 3 | 1.5 | 12.5 |
| Kim et al, | 2009 | 1 | 1 | 3 | 3 | 3 | 1.5 | 12.5 |
| Kramp et al, | 2015 | 1 | 1 | 3 | 3 | 3 | 1.5 | 12.5 |
| Kramp et al, | 2016 | 1 | 1 | 3 | 3 | 3 | 1.5 | 12.5 |
| Larson et al, | 2005 | 1 | 1 | 3 | 3 | 3 | 1.5 | 12.5 |
| Lavanchy et al, | 2021 | 1 | 1 | 3 | 3 | 3 | 1.5 | 12.5 |
| Leifman et al, | 2023 | 1 | 2 | 3 | 2 | 3 | 1.5 | 12.5 |
| Steigerwald et al, | 2015 | 1 | 1 | 3 | 3 | 3 | 1.5 | 12.5 |
| van Zwieten et al, | 2021 | 1 | 2 | 3 | 2 | 3 | 1.5 | 12.5 |
| Williams et al | 2012 | 1 | 1 | 3 | 3 | 3 | 1.5 | 12.5 |
| Williams et al | 2015 | 1 | 1 | 3 | 3 | 3 | 1.5 | 12.5 |
| Wohaibi et al, | 2007 | 1 | 1 | 3 | 3 | 3 | 1.5 | 12.5 |
| Adrales et al | 2024 | 1 | 1.5 | 3 | 2 | 3 | 1.5 | 12 |
| Aggarwal et al, | 2008 | 1 | 1.5 | 3 | 3 | 2 | 1.5 | 12 |
| Aggarwal et al, | 2007 | 1 | 1.5 | 3 | 3 | 2 | 1.5 | 12 |
| Dosis et al, | 2005 | 1 | 1.5 | 3 | 2 | 3 | 1.5 | 12 |
| Pradeep and Sinha | 2023 | 1 | 1.5 | 3 | 2 | 3 | 1.5 | 12 |
| Sarker et al, | 2006a | 1 | 1.5 | 3 | 2 | 3 | 1.5 | 12 |
| Yanagida et al, | 2025 | 1 | 1.5 | 3 | 2 | 3 | 1.5 | 12 |
| Adrales et al | 2024 | 1 | 1.5 | 3 | 2 | 3 | 1 | 11.5 |
| Balvardi et al, | 2022 | 1 | 1 | 3 | 2 | 3 | 1.5 | 11.5 |
| Chevallay et al, | 2021 | 1 | 1 | 3 | 2 | 3 | 1.5 | 11.5 |
| Choy et al, | 2012 | 1 | 1 | 3 | 2 | 3 | 1.5 | 11.5 |
| Ganni et al, | 2020 | 1 | 1 | 3 | 2 | 3 | 1.5 | 11.5 |
| Hwang et al | 2006 | 1 | 1 | 3 | 2 | 3 | 1.5 | 11.5 |
| Kawamura et al, | 2023 | 1 | 1 | 3 | 2 | 3 | 1.5 | 11.5 |
| Mascagani et al, | 2022 | 1 | 1 | 3 | 2 | 3 | 1.5 | 11.5 |
| Petrachi et al, | 2024 | 1 | 2 | 3 | 2 | 2 | 1.5 | 11.5 |
| Quick et al, | 2017 | 1 | 1 | 3 | 2 | 3 | 1.5 | 11.5 |
| Sarker et al, | 2005 | 1 | 1 | 3 | 2 | 3 | 1.5 | 11.5 |
| Scott et al, | 2000a | 1 | 1 | 3 | 2 | 3 | 1.5 | 11.5 |
| Scott et al, | 2000b | 1 | 1 | 3 | 2 | 3 | 1.5 | 11.5 |
| Szasz et al, | 2017b | 1 | 1 | 3 | 2 | 3 | 1.5 | 11.5 |
| Yen et al, | 2025 | 1 | 1 | 3 | 2 | 3 | 1.5 | 11.5 |
| Ganni et al, | 2018 | 1 | 1 | 3 | 2 | 2 | 1.5 | 10.5 |
| Gumbs et al, | 2007 | 1 | 1 | 3 | 2 | 2 | 1.5 | 10.5 |
| Jin et al, | 2018 | 1 | 1 | 3 | 2 | 2 | 1.5 | 10.5 |
| Seymour et al, | 2003 | 1 | 1 | 3 | 2 | 2 | 1.5 | 10.5 |
| Steiman et al, | 2018 | 1 | 1 | 3 | 2 | 2 | 1.5 | 10.5 |
| Tang et al, | 2004 | 1 | 1 | 3 | 2 | 2 | 1.5 | 10.5 |
| Isreb et al, | 2020 | 1 | 2 | 3 | 1 | 2 | 1 | 10 |
| Joice et al, | 1998 | 1 | 1 | 3 | 1 | 2 | 1.5 | 9.5 |
