# Supplementary material for: Objective technical skills assessment in laparoscopic cholecystectomy: a systematic review of manual, kinematic, and AI-based tools
Source: Surg Endosc. 2026 Aug 31;40(10):8285–303. doi: 10.1007/s00464-026-13309-z (PMC13633379; doi:10.1007/s00464-026-13309-z)
Supplement: Supplementary file 7 [file 464_2026_13309_MOESM7_ESM.docx]

**Supplementary Table S6. COSMIN risk-of-bias assessment of manual technical skills assessment studies in laparoscopic cholecystectomy.** Manual assessment studies were appraised using the COnsensus-based Standards for the selection of health Measurement INstruments (COSMIN) Risk of Bias framework according to the measurement properties evaluated in each study. Methodological quality was rated as very good, adequate, doubtful, or inadequate, with overall ratings determined according to COSMIN criteria.

| **KEY** | Very good (VG) | Adequate (A) | Doubtful (D) | Inadequate (I) |
| --- | --- | --- | --- | --- |

| **AUTHOR/YEAR** | **SCORE** | | | | | | | | | | |
| --- | --- | --- | --- | --- | --- | --- | --- | --- | --- | --- | --- |
| Adrales 2024 | A | A |  |  |  | I |  |  |  |  | **I** |
| Adrales 2024 | A | A |  |  |  |  |  |  |  |  | **A** |
| Aggarwal 2008 |  |  |  | VG |  | VG |  |  | VG |  | **VG** |
| Balvardi 2022 |  |  |  |  |  |  |  |  | VG |  | **VG** |
| Chang 2007 |  |  |  |  |  |  |  |  | D |  | **D** |
| Chevallay 2021 | I | I |  |  |  | VG |  |  | A |  | **I** |
| Choy 2012 |  |  |  |  |  | VG |  |  |  |  | **VG** |
| Eubanks 1999 |  |  |  |  |  | A |  |  | A |  | **A** |
| Gumbs 2007 |  |  |  |  |  |  |  |  | VG |  | **VG** |
| Hopmans 2014 |  |  |  | VG |  |  |  |  | VG |  | **VG** |
| Huber 2018 | I |  |  | VG |  | VG |  |  | VG |  | **I** |
| Humm 2023 |  |  |  |  |  | VG |  |  | A |  | **A** |
| Isreb 2020 |  |  |  |  |  |  |  |  | D |  | **D** |
| Joice 1998 | D | D |  |  |  |  |  |  |  |  | **D** |
| Kavaluri 2025 |  |  |  |  |  | VG |  |  | VG | A | **A** |
| Kim 2009 |  |  |  | D |  | VG |  |  | A |  | **D** |
| Kramp 2015 |  |  |  | I |  | VG |  |  | VG | VG | **I** |
| Kramp 2016 | A | A |  | I |  | VG |  |  | VG |  | **I** |
| Lam 2014 |  |  |  |  |  | VG |  |  |  |  | **VG** |
| Larson 2005 | D |  |  |  |  | I |  |  | D |  | **I** |

| **AUTHOR/YEAR** | **SCORE** | | | | | | | | | | |
| --- | --- | --- | --- | --- | --- | --- | --- | --- | --- | --- | --- |
| Malik 2016 |  |  |  | VG |  | VG |  |  | A |  | **A** |
| Quick 2017 |  |  |  |  |  | VG |  |  | A |  | **A** |
| Sarker 2006a | D |  |  |  |  | VG |  |  |  |  | **D** |
| Sarker 2010 |  |  |  |  |  | VG |  |  | A |  | **A** |
| Sarker 2006c | D |  |  |  |  | VG |  |  |  |  | **D** |
| Sarker 2006b | D |  |  |  |  | VG |  |  |  |  | **D** |
| Sarker 2005 | D |  |  |  |  | VG |  |  |  |  | **D** |
| Scott 2000a |  |  |  |  |  | A |  |  | VG |  | **A** |
| Scott 2000b |  |  |  |  |  |  |  |  | A |  | **A** |
| Seymour 2003 | I |  |  |  |  | I |  |  |  |  | **I** |
| Steigerwald 2015 |  |  |  | VG |  |  |  | VG | VG |  | **VG** |
| Steiman 2018 |  |  |  |  |  |  |  |  | D |  | **D** |
| Szasz 2017a |  |  |  |  |  | VG |  |  | VG |  | **VG** |
| Szasz 2017b |  |  |  |  |  | I |  |  | A |  | **I** |
| Tang 2004 |  | D |  |  |  |  |  |  |  |  | **D** |
| van Zweiten 2021 |  |  |  |  |  |  |  |  | VG |  | **VG** |
| Vassiliou 2007 |  |  |  |  |  | VG |  |  | VG |  | **VG** |
| Vassiliou 2005 | D |  |  | VG |  | VG |  |  | VG |  | **D** |
| Williams 2012 |  |  |  | VG |  |  | A | A | A |  | **A** |
| Williams 2015 |  |  |  |  |  | VG |  | VG | A |  | **A** |
| Wohaibi 2007 |  | I |  | VG |  |  |  |  | A |  | **A** |

**Overall score**

**Responsiveness**

**Construct validity**

**Criterion validity**

**Measurement error**

**Reliability**

**Cross cultural validity**

**Internal consistency**

**Structural validity**

**Content validity**

**PROM development**

COSMIN = COnsensus-based Standards for the selection of health Measurement INstruments; LC = laparoscopic cholecystectomy
