# Supplementary material for: Objective technical skills assessment in laparoscopic cholecystectomy: a systematic review of manual, kinematic, and AI-based tools
Source: Surg Endosc. 2026 Aug 31;40(10):8285–303. doi: 10.1007/s00464-026-13309-z (PMC13633379; doi:10.1007/s00464-026-13309-z)
Supplement: Supplementary file 8 [file 464_2026_13309_MOESM8_ESM.docx]

**Supplementary Table S7. COSMIN risk-of-bias assessment of kinematic technical skills assessment studies in laparoscopic cholecystectomy.** Kinematic assessment studies were appraised using the COnsensus-based Standards for the selection of health Measurement INstruments (COSMIN) Risk of Bias framework according to the measurement properties evaluated in each study. Methodological quality was rated as very good, adequate, doubtful, or inadequate, with overall ratings determined according to COSMIN criteria.

| **KEY** | Very good (VG) | Adequate (A) | Doubtful (D) | Inadequate (I) |
| --- | --- | --- | --- | --- |

| **AUTHOR/YEAR** | **SCORE** | | | | | | | | | | |
| --- | --- | --- | --- | --- | --- | --- | --- | --- | --- | --- | --- |
| Aggarwal 2007 |  |  |  |  |  | I |  |  | VG |  | **I** |
| Cristancho 2008 |  | I |  |  |  | I |  |  | VG |  | **I** |
| Dosis 2005 |  |  |  |  |  |  |  |  | D |  | **D** |
| Ganni 2018 |  | I |  |  |  |  |  |  | VG |  | **I** |
| Ganni 2020 |  |  |  |  |  |  |  |  | D |  | **D** |
| Hwang 2006 |  |  |  |  |  |  |  |  | D |  | **D** |

**Overall score**

**Responsiveness**

**Construct validity**

**Criterion validity**

**Measurement error**

**Reliability**

**Cross cultural validity**

**Internal consistency**

**Structural validity**

**Content validity**

**PROM development**

COSMIN = COnsensus-based Standards for the selection of health Measurement INstruments; LC = laparoscopic cholecystectomy.
