# Supplementary material for: Objective technical skills assessment in laparoscopic cholecystectomy: a systematic review of manual, kinematic, and AI-based tools
Source: Surg Endosc. 2026 Aug 31;40(10):8285–303. doi: 10.1007/s00464-026-13309-z (PMC13633379; doi:10.1007/s00464-026-13309-z)
Supplement: Supplementary file 9 [file 464_2026_13309_MOESM9_ESM.docx]

**Supplementary Table S8. QUADAS-2 risk-of-bias and applicability assessment of AI-based technical skills assessment studies in laparoscopic cholecystectomy.** AI-based studies were appraised using the Quality Assessment of Diagnostic Accuracy Studies-2 (QUADAS-2) framework across the patient selection, index test, reference standard, and flow and timing domains. Risk of bias was rated as low, high, or unclear, and applicability concerns were assessed for patient selection, the index test, and the reference standard.

| **KEY** | Low risk ☺ | High risk ☹ | Unclear ? |
| --- | --- | --- | --- |

| **STUDY** | **RISK OF BIAS** | | | | **APPLICABILITY CONCERNS** | | |
| --- | --- | --- | --- | --- | --- | --- | --- |
| **Author/Year** | **Patient selection** | **Index test** | **Reference standard** | **Flow and timing** | **Patient selection** | **Index test** | **Reference standard** |
| Chen 2024 | ☹ | ☺ | ☺ | ☺ | ☺ | ☺ | ☺ |
| Humm 2023 | ☹ | ☺ | ☹ | ☺ | ☺ | ☺ | ☹ |
| Jin 2019 | ? | ☺ | ☺ | ☺ | ☺ | ☺ | ☺ |
| Kawamura 2023 | ☹ | ☺ | ☺ | ☺ | ☺ | ☺ | ☺ |
| Lavanchy 2021 | ☹ | ☹ | ☺ | ☺ | ☹ | ☹ | ☺ |
| Leifman 2023 | ? | ☺ | ☺ | ☺ | ☺ | ☺ | ☺ |
| Mascagni 2022 | ☺ | ☺ | ☺ | ☺ | ☺ | ☺ | ☺ |
| Petracchi 2024 | ? | ☺ | ☺ | ☺ | ☺ | ☺ | ☺ |
| Pradeep 2023 | ? | ☺ | ☺ | ☹ | ☺ | ☺ | ☺ |
| Wu 2023 | ☺ | ☺ | ☺ | ☺ | ☺ | ☺ | ☺ |
| Yanagida 2025 | ☺ | ☺ | ☺ | ☺ | ☺ | ☺ | ☺ |
| Yanik 2024 | ? | ☺ | ? | ☺ | ☺ | ? | ? |
| Yen 2025 | ? | ☺ | ☺ | ☺ | ☺ | ☺ | ☺ |

AI = artificial intelligence; QUADAS-2 = Quality Assessment of Diagnostic Accuracy Studies-2; LC = laparoscopic cholecystectomy.
